# Supplementary material for: Correction of a urea cycle defect after ex vivo gene editing of human hepatocytes
Source: Mol Ther. 2021 Jan 21;29(5):1903–17. doi: 10.1016/j.ymthe.2021.01.024 (PMC8116578; doi:10.1016/j.ymthe.2021.01.024)
Supplement: Document S1. Figures S1–S8 and Tables S1 and S2 [file mmc1.pdf]

YMTHE, Volume *29*

Supplemental Information

Correction of a urea cycle defect after *ex vivo* gene editing of human hepatocytes

Mihaela Zabulica, Raghuraman C. Srinivasan, Pinar Akcakaya, Gabriella Allegri, Burcu Bestas, Mike Firth, Christina Hammarstedt, Tomas Jakobsson, Towe Jakobsson, Ewa Ellis, Carl Jorns, Georgios Makris, Tanja Scherer, Nicole Rimann, Natalie R. van Zuydam, Roberto Gramignoli, Anna Forslöw, Susanna Engberg, Marcello Maresca, Olav Rooyackers, Beat Thöny, Johannes Häberle, Barry Rosen, and Stephen C. Strom

Supplemental Figures

A

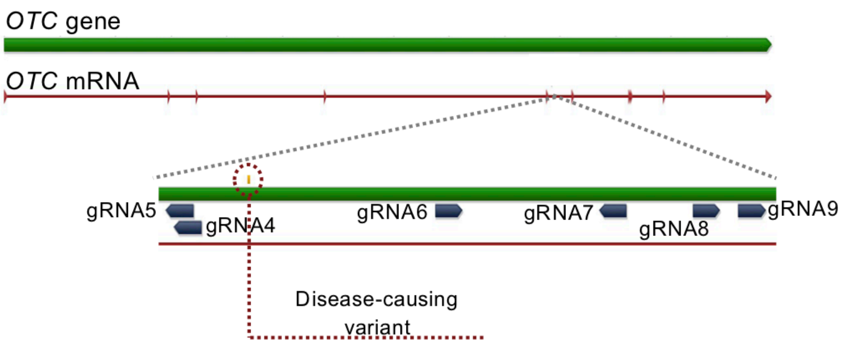

B

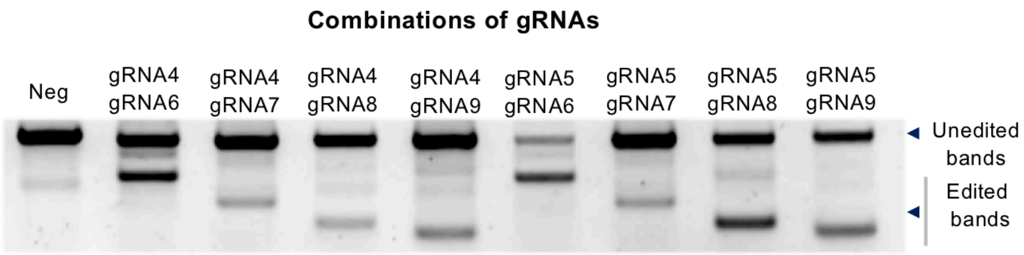

Supplemental Figure S1: Screening of combinations of gRNAs for efficient cleavage of the ectopic splice site. (A) Genomic structure representation of *OTC* gene (green) and transcript (red), and the position of CRISPR gRNAs tested. Two alternatives of gRNAs are positioned upstream (gRNA4 and gRNA5), and four gRNAs downstream (gRNA6, gRNA7, gRNA8 and gRNA9) of the pathogenic variant, and are used pairwise. **(B)** Estimation of editing efficiency of different pairs of gRNAs by PCR amplification of the target region. Upper and lower amplicon bands show unedited (wild-type) and edited bands, respectively, and are indicated with arrows. Neg: Non-transfected cells.

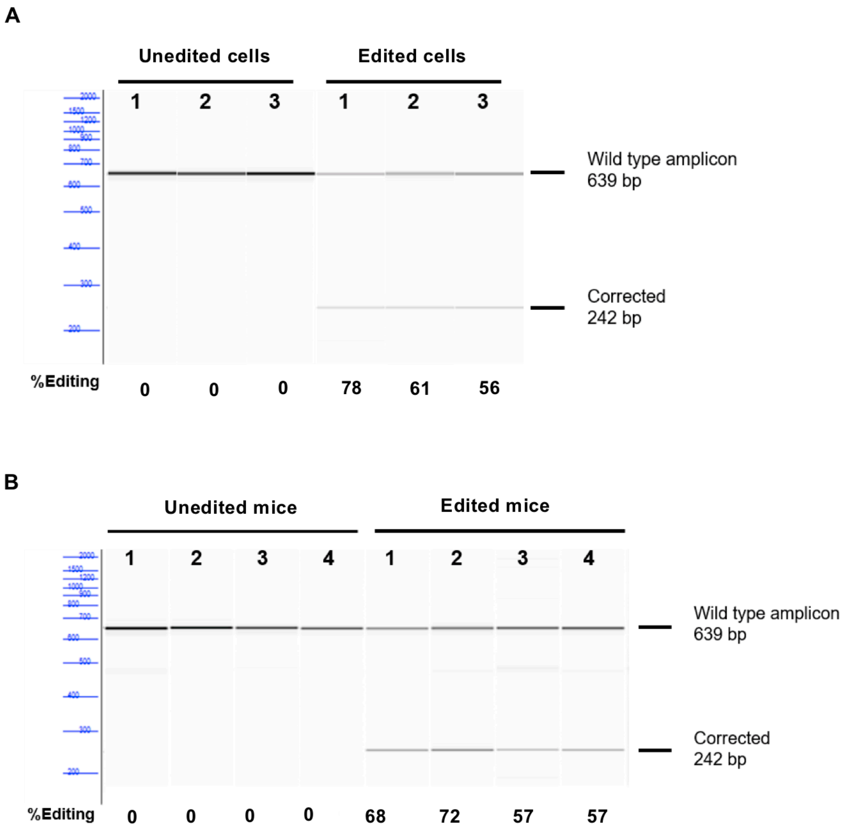

Supplemental Figure S2: gRNA5-gRNA8 dual guides with *Sp*Cas9 efficiently mutates *OTC* gene in patient-derived primary hepatocytes and humanized mouse livers. (A) Editing was evaluated in n = 3 transfection replicates (labelled as 1, 2 and 3) using genomic DNA isolated from the cells electroporated with dual RNP complex gRNA5-Cas9 and gRNA8–Cas9, and unedited OTCD cells electroporated with only Cas9 enzyme. Assay was performed 48 hours following electroporation. **(B)** Editing was evaluated in n = 4 biological replicates (labelled as 1, 2, 3 and 4) using genomic DNA isolated from the humanized mouse livers transplanted with corrected patient hepatocytes edited by dual RNP complex gRNA5-Cas9 and gRNA8–Cas9, and unedited OTCD cells. Assay was performed when mice reached desired repopulation levels. Gene region was amplified with sequence specific primers. Wild type (639 bp) and mutated (242 bp) amplicons were quantified using fragment analyzer.

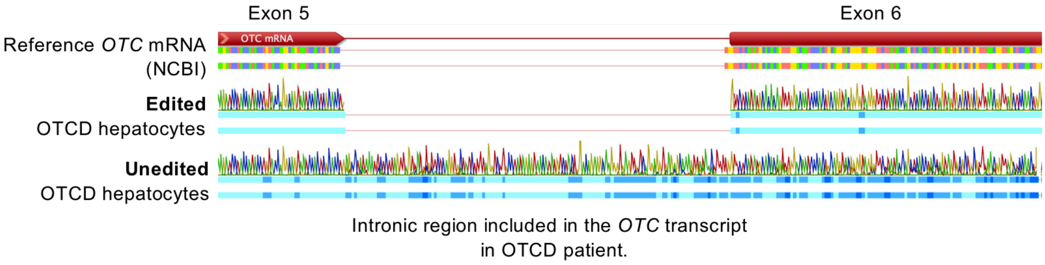

Supplemental Figure S3: Sequencing of *OTC* transcript in edited and unedited OTC deficient (OTCD) hepatocytes. *OTC* transcripts in genetically edited and unedited patient OTCD hepatocytes were amplified, sequenced and aligned to reference *OTC* transcript (NCBI NP_000522.3). The intronic region included between exons 5 and 6 in the mutated transcript in patient OTCD hepatocytes is absent in OTCD edited cells.

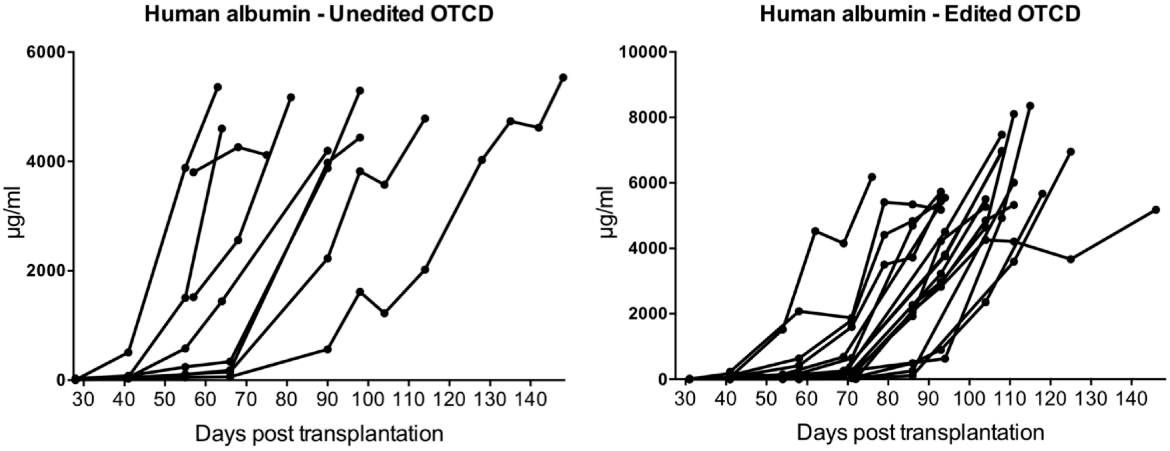

Supplemental Figure S4: Human albumin levels at different time points post transplantation in Unedited OTC deficient (OTCD) and Edited OTCD groups. Each line represents an experimental animal. Unedited OTCD mice n=9. Edited OTCD mice n=18.

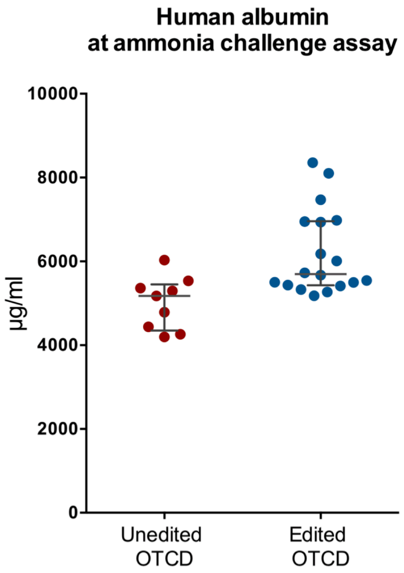

Supplemental Figure S5: Human albumin levels at ammonia challenge. Human albumin measurements when mice were subjected to ammonia challenge assay *in vivo.* Unedited OTCD mice n=9. Edited OTCD mice n=18.

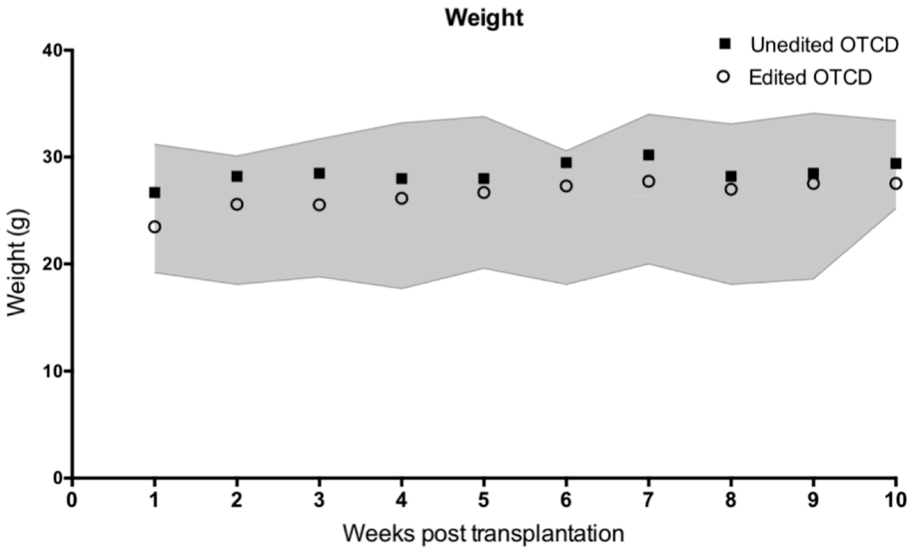

Supplemental Figure S6: Weight. Median weight of experimental groups that were either liver-humanized with edited or unedited patient OTC deficient (OTCD) hepatocytes every week post transplantation. Grey-highlighted area represents weight range of healthy controls (mice repopulated with OTC proficient hepatocytes).

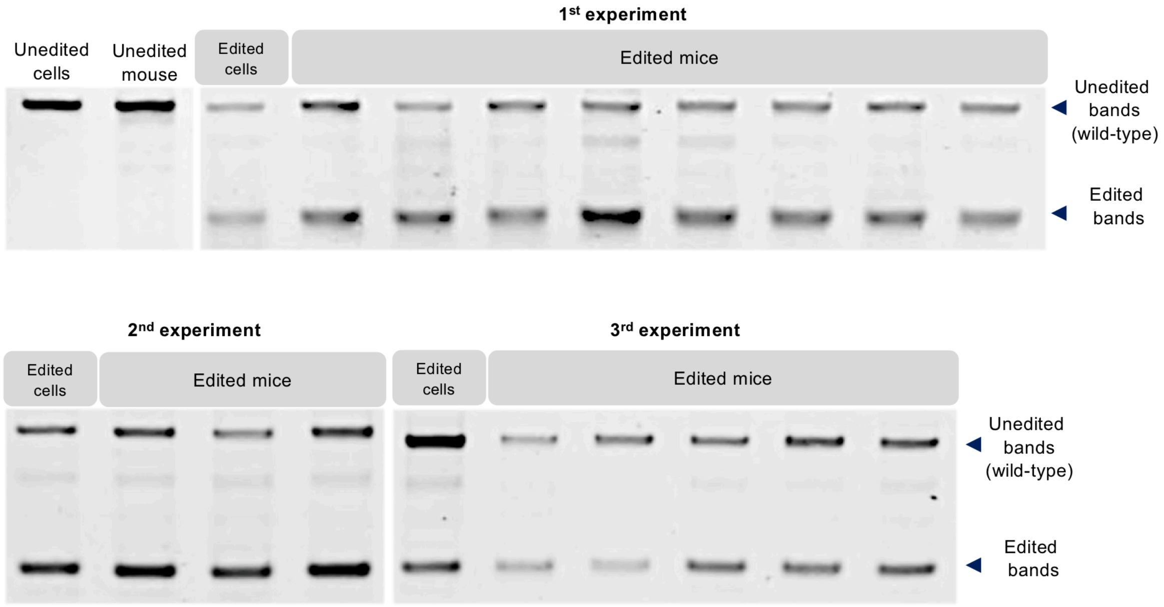

Supplemental Figure S7: Estimation of editing efficiency in hepatocytes prior transplantation and in liver-humanized mice with the respective cells. PCR amplification of target region in the *OTC* gene in OTC deficient (OTCD, either edited or unedited) hepatocytes prior transplantation and in humanized livers with the respective cells from three independent experiments. Upper and lower band indicate unedited (wild-type) and edited DNA, respectively. Actual editing efficiency was estimated based on band intensity and amplicon length, and shown in Figure 3A. Unedited cells: OTCD hepatocytes not genetically engineered. Unedited mouse: Humanized mouse liver with unedited OTCD hepatocytes. Edited cells: OTCD hepatocytes genetically engineered and used for each experiment. Edited mice: Humanized mouse livers with edited OTCD hepatocytes (each lane corresponds to DNA from a different humanized mouse liver).

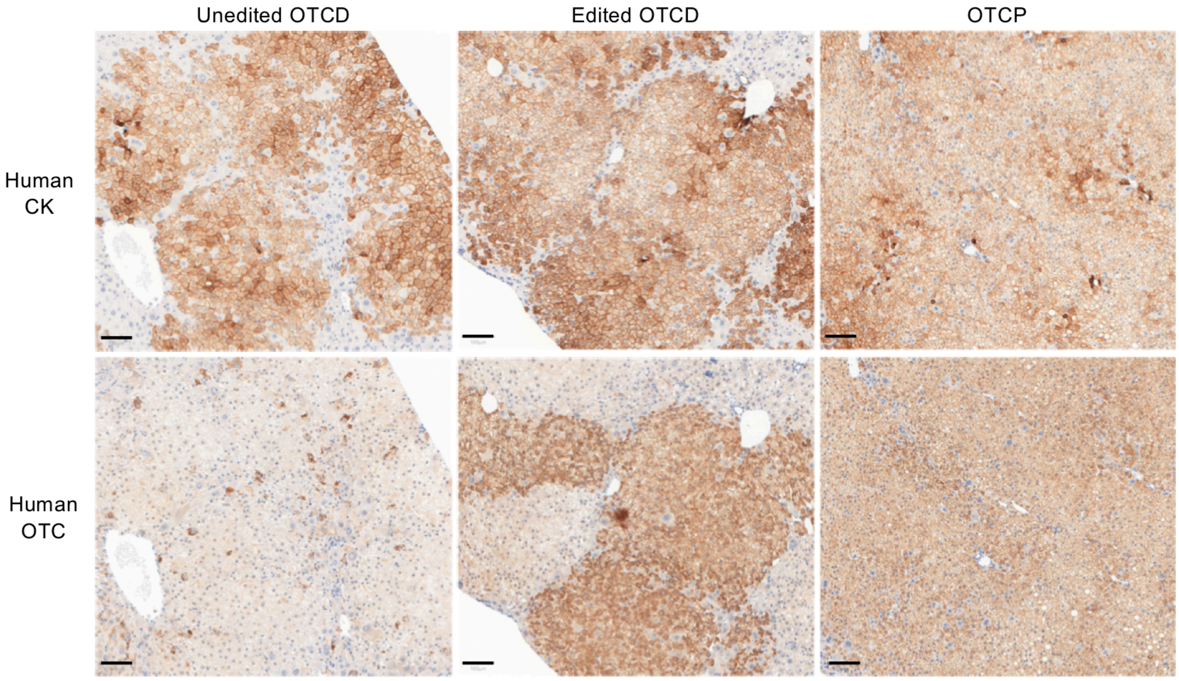

Supplemental Figure S8: Immunohistochemical analyses of liver tissue from liver-humanized mice. Representative sections of mouse liver immunostained with antibodies against human CK or OTC in mice that received either unedited or edited OTC deficient (OTCD), or OTC proficient (OTCP) human hepatocytes. Areas positive for CK indicate the areas repopulated with human hepatocytes. Positive reaction with antibodies to OTC indicates cells that express full-length, normal protein. Scale bar 100 µm.

Supplemental Tables

Supplemental Table 1: Sequence and position of gRNA tested for optimizing editing efficiency.

gRNA name	5' - sequence - 3'
gRNA4	GATATGGGCTAAAAGGGTTT
gRNA5	GGCTAAAAGGGTTTGGGAAT
gRNA6	GGTTAGTTACTAAGTCACTC
gRNA7	CTAATTTGGGAGGACTAGAA
gRNA8	AATCTACATTATATTGCCCC
gRNA9	AGCCATGGTTTCTATGTCTA

Position of gRNAs tested:

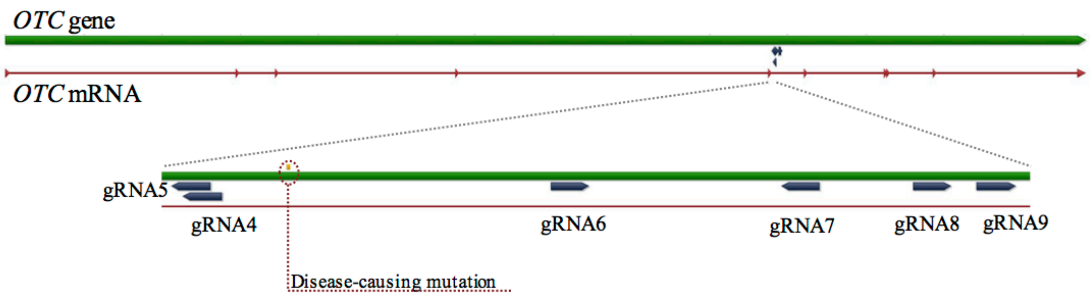

Supplemental Table 2: TaqMan assays used for gene expression analyses.

	Gene name	Gene full name	Assay ID
	PPIA	Cyclophilin A (peptidylprolyl isomerase A)	Hs99999904_m1
**Urea cycle genes**	*OTC*	Ornithine carbamoyltransferase	Hs00166892_m1
	CPS1	Carbamoyl-phosphate synthetase 1, mitochondrial	Hs00157048_m1
	ASS1	Argininosuccinate synthetase	Hs01597989_g1
	ASL	Argininosuccinate lyase	Hs00902699_m1
	ARG1	Arginase 1	Hs00163660_m1
**Liver proteins and transcription factors**	*ALB*	Albumin	Hs00609411_m1
	AFP	Alpha-fetoprotein	Hs00173490_m1
	FAH	Fumarylacetoacetate hydrolase	Hs00908445_m1
	A1AT	Alpha-1 antitrypsin (SERPINA1)	Hs01097800_m1
	HNF4a	Hepatic nuclear factor 4 alpha	Hs0102395_m1
	HNF6	Hepatic nuclear factor 6 (ONECUT1)	Hs00413554_m1
	CAR	Nuclear receptor subfamily 1, I3 (NR1I3)	Hs00901570_g1
	PXR	Nuclear receptor subfamily 1, I2 (NR1I2)	Hs01114267_m1
**Phase I genes**	*CYP1A1*	Cytochrome P450 family 1 subfamily A member 1	Hs00153120_m1
	CY1A2	Cytochrome P450 family 1 subfamily A member 2	Hs01070374_m1
	CYP2B6	Cytochrome P450 family 2 subfamily B member 6	Hs03044634_m1
	CYP2C8	Cytochrome P450 family 2 subfamily C member 8	Hs00258314_m1
	CYP2C9	Cytochrome P450 family 2 subfamily C member 9	Hs00426397_m1
	CYP2C19	Cytochrome P450 family 2 subfamily C member 19	Hs00426380_m1
	CYP3A4	Cytochrome P450 family 3 subfamily A member 4	Hs00430021_m1
	CYP3A7	Cytochrome P450 family 3 subfamily A member 7	Hs00426361_m1
**Phase II genes**	*UGT1A1*	UDP glucuronosyltransferase 1 family, A1	Hs02511055_s1
	UGT1A6	UDP glucuronosyltransferase 1 family, A6	Hs01592477_m1
	UGT1A9	UDP glucuronosyltransferase 1 family, A9	Hs02516855_sH
	UGT2B7	UDP glucuronosyltransferase 2 family, B7	Hs00426592_m1
	UGT2B17	UDP glucuronosyltransferase 2 family, B17	Hs00854486_sH
**Transporters**	*MRP2*	ATP-binding cassette, C2 (ABCC2)	Hs00166123_m1
	MRP3	ATP-binding cassette, C3 (ABCC3)	Hs00978473_m1
	MRP4	ATP-binding cassette, C4 (ABCC4)	Hs00988717_m1
	BSEP	ATP-binding cassette, B11 (ABCB11)	Hs00184824_m1
	BCRP	ATP-binding cassette, G2 (ABCG2)	Hs00184979_m1
	NTCP	Sodium/bile acid cotransporter 1 (SLC10A1)	Hs00914889_m1

Supplemental Table 3: Provided as a separate Excel file.
Off-target sites identified by CIRCLE-seq for gRNA5 and Cas9 nuclease. Chromosomal coordinates are listed, followed by the CIRCLE-seq read count for each site for two replicates, the off-target sequence, number of mismatches, bulge mismatch sequence, score for the off-targets with bulges, and annotations according to Ensembl as gene and region.

Supplemental Table 4: Provided as a separate Excel file.
Off-target sites identified by CIRCLE-seq for gRNA8 and Cas9 nuclease. Chromosomal coordinates are listed, followed by the CIRCLE-seq read count for each site for two replicates, the off-target sequence, number of mismatches, bulge mismatch sequence, score for the off-targets with bulges, and annotations according to Ensembl as gene and region.

Supplemental Table 5: Provided as a separate Excel file.
Potential off-target sites for gRNA5 and Cas9, analysed by targeted amplicon deep sequencing in primary hepatocytes. Samples are organized according to treatment status and replicate number. For each off-target site, chromosomal coordinates are listed, followed by columns detailing the read counts for each sample, median percentage of indels for each treatment group and statistical test results between the edited and control groups, followed by primers used. Coef_flag: Nuclease-treatment coefficient, freq_flag: median indel frequency of edited samples and sig_flag: adjusted p value are evaluated to determine off-targets reported in the off-target column.

Supplemental Table 6: Provided as a separate Excel file.
Potential off-target sites for gRNA8 and Cas9, analyzed by targeted amplicon deep sequencing in primary hepatocytes. Samples are organized according to treatment status and replicate number. For each off-target site, chromosomal coordinates are listed, followed by columns detailing the read counts for each sample, median percentage of indels for each treatment group and statistical test results between the edited and control groups, followed by primers used. Coef_flag: Nuclease-treatment coefficient, freq_flag: median indel frequency of edited samples and sig_flag: adjusted p value are evaluated to determine off-targets reported in the off-target column.
