# Supplementary material for: Correction of a urea cycle defect after ex vivo gene editing of human hepatocytes
Source: Mol Ther. 2021 Jan 21;29(5):1903–17. doi: 10.1016/j.ymthe.2021.01.024 (PMC8116578; doi:10.1016/j.ymthe.2021.01.024)
Supplement: Document S2. Article plus supplemental information [file mmc6.pdf]

Molecular Therapy
Original Article

Correction of a urea cycle defect after *ex vivo* gene editing of human hepatocytes

Mihaela Zabulica,[1] Raghuraman C. Srinivasan,[1] Pinar Akcakaya,[2] Gabriella Allegri,[3] Burcu Bestas,[2] Mike Firth,[4] Christina Hammarstedt,[1] Tomas Jakobsson,[1] Towe Jakobsson,[5] Ewa Ellis,[5] Carl Jorns,[5] Georgios Makris,[3] Tanja Scherer,[3] Nicole Rimann,[3] Natalie R. van Zuydam,[6] Roberto Gramignoli,[1] Anna Forslöw,[2] Susanna Engberg,[2] Marcello Maresca,[2] Olav Rooyackers,[5] Beat Thöny,[3] Johannes Häberle,[3] Barry Rosen,[4] and Stephen C. Strom[1]

[1]Department of Laboratory Medicine, Karolinska Institutet, 141 52 Huddinge, Sweden; [2]Discovery Sciences, BioPharmaceuticals R&D Unit, AstraZeneca, Gothenburg, Sweden; [3]Division of Metabolism and Children's Research Center, University Children's Hospital, Zürich, Switzerland; [4]Discovery Sciences, BioPharmaceuticals R&D Unit, AstraZeneca, Cambridge, UK; [5]Department of Clinical Sciences Intervention and Technology, Karolinska Institutet, Stockholm, Sweden; [6]Department of Quantitative Biology, Discovery Sciences, R&D BioPharmaceuticals, AstraZeneca, Gothenburg, Sweden

Ornithine transcarbamylase deficiency (OTCD) is a monogenic disease of ammonia metabolism in hepatocytes. Severe disease is frequently treated by orthotopic liver transplantation. An attractive approach is the correction of a patient's own cells to regenerate the liver with gene-repaired hepatocytes. This study investigates the efficacy and safety of *ex vivo* correction of primary human hepatocytes. Hepatocytes isolated from an OTCD patient were genetically corrected *ex vivo*, through the deletion of a mutant intronic splicing site achieving editing efficiencies >60% and the restoration of the urea cycle *in vitro*. The corrected hepatocytes were transplanted into the liver of FRGN mice and repopulated to high levels (>80%). Animals transplanted and liver repopulated with genetically edited patient hepatocytes displayed normal ammonia, enhanced clearance of an ammonia challenge and OTC enzyme activity, as well as lower urinary orotic acid when compared to mice repopulated with unedited patient hepatocytes. Gene expression was shown to be similar between mice transplanted with unedited or edited patient hepatocytes. Finally, a genome-wide screening by performing CIRCLE-seq and deep sequencing of >70 potential off-targets revealed no unspecific editing. Overall analysis of disease phenotype, gene expression, and possible off-target editing indicated that the gene editing of a severe genetic liver disease was safe and effective.

INTRODUCTION

The urea cycle, consisting of six enzymes and two transporters, is localized mainly in the liver and is essential for arginine biosynthesis and the metabolism of ammonia to urea for elimination from the body. Mutations in any of the urea cycle genes can lead to urea cycle disorders (UCDs). The most frequent UCD is ornithine transcarbamylase deficiency (OTCD), which is X-linked and has an incidence of 1:56,500.[1] If left untreated, severe mutations in *OTC* can lead to life-threatening episodes of hyperammonemia and central nervous system damage in the neonatal period and throughout life. The standard of care is a protein-restricted diet supplemented with ammonia scavengers, but these measures are not always sufficient to maintain patient health, metabolic stability, and normal ammonia levels. The definitive treatment of severe UCD is orthotopic liver transplantation. This and other studies indicate that allogeneic hepatocyte transplantation can provide only partial and temporary corrections,[2–4] and recent reports suggest that immune-mediated rejection of the donor cells may limit the survival of donor hepatocytes.[5,6] Direct correction of the mutation in a patient's own hepatocytes would likely avoid the need for immunosuppressive drugs and immune rejection of cell grafts. Preclinical studies of the gene editing of mutations in human hepatocytes are needed to optimize efficiency and investigate the safety of the technology. Recent developments in genome engineering, specifically CRISPR (clustered regularly interspaced short palindromic repeats) technology, have renewed interest in the treatment of genetic disorders through precise gene editing procedures both *ex vivo* and *in vivo*.[7–9] Autologous and allogeneic cell therapies employing *ex vivo* CRISPR genome engineering have recently entered clinical trials for hematopoietic stem cell (HSC) transplants to treat beta globinopathies (ClinicalTrials.gov: NCT03655678 and NCT03745287) and chimeric antigen receptor (CAR) T-based anti-cancer therapies of the immune system (ClinicalTrials.gov: NCT03398967, NCT03398967, and NCT0369011).

Studies have attempted CRISPR-mediated correction of mutations in genes that cause liver diseases, such as hereditary tyrosinemia,[10,11] a urea cycle defect,[12] and phenylketonuria.[13] However, these studies have been performed on mouse cells or in mice. Questions remain as to whether preclinical therapeutic genome editing studies performed in rodents can be confidently translated to human patients due to significant differences between the species in genomic sequences and DNA repair pathways. Target cell humanized chimeric

Received 12 July 2020; accepted 12 January 2021;
https://doi.org/10.1016/j.ymthe.2021.01.024.

Correspondence: Stephen C. Strom, PhD, Department of Laboratory Medicine, Karolinska Institutet, Alfred Nobels Allé 8, 141 52 Huddinge, Sweden.
E-mail: stephen.strom7.4@gmail.com

This is an open access article under the CC BY license (http://creativecommons.org/licenses/by/4.0/).

experimental animals are valuable models to evaluate the safety and efficiency of therapeutic genome engineering. A chimeric model for liver humanization is the FRGN mouse (*Fah*$^{-/-}$, *Rag2*$^{-/-}$, and *Il2rg*$^{-/-}$ on the NOD-strain background), that is severely immunocompromised and conditionally hepatocompromised, so that it enables efficient repopulation of the liver with up to 95% donor human hepatocytes to form a functional liver.[14,15] A recent report indicated that liver-humanized mice with a different UCD, carbamoyl phosphate synthetase 1 (CPS1) deficiency, effectively reproduced the human disease phenotype.[16] Here, we report the creation of a liver-humanized mouse model of OTCD by the transplantation of human hepatocytes derived from a patient with severe OTCD. In addition, when liver-humanized mice were generated by the transplantation of OTCD hepatocytes after *ex vivo* genetic correction of the disease-causing mutation, the mice displayed a significant phenotypic correction of disease symptoms. Analysis of gene expression, liver function, and possible off-target editing indicated that the treatment was safe and effective. In addition, gene-edited hepatocytes could robustly proliferate and efficiently repopulate the liver. This work provides evidence of the efficacy of CRISPR editing in human hepatocytes and establishes a model system with which the safety of these procedures can be evaluated *in vivo* for long periods of time.

RESULTS

Pseudoexon inclusion caused by an OTC mutation in patient hepatocytes

Hepatocytes were isolated and cryopreserved from the donated liver of a male patient undergoing a liver transplant due to severe OTCD. As *OTC* is an X-linked locus, the patient cells contain a single copy of the mutant gene and no wild-type copies. To identify the specific mutation in this patient, the *OTC* transcript was analyzed from patient-derived hepatocytes (denoted as OTCD) with comparison to normal hepatocytes (denoted as OTC proficient, OTCP) by RT-PCR. The amplified product covering exons 1–5 was identical between the OTCD case and OTCP hepatocytes, while the amplified transcript spanning exons 5–10 had 2 different lengths, a minor band of wild-type length and a major band, were ~130–140 nt longer than the wild-type transcript (Figure 1A). DNA sequencing of the intronic region revealed a single nucleotide substitution at c.540+265G > A, which generates a novel splice acceptor site within intron 5 (Figure 1B). The mutation leads to the insertion of 135 nt of a normally intronic region into the *OTC* coding transcript and generates a premature stop codon shortly after exon 5 in the OTC protein (p.Q180_E181insCHI*) (Figure 1C). This mutation has been previously reported in another severely affected OTCD male and would be predicted to be a severe loss of function phenotype, which abolishes OTC enzyme activity.[17]

Corrective CRISPR genome editing restores urea cycle activity in OTCD hepatocytes *in vitro*

Since no mutations were detected in exonic sequences, it is predicted that the deletion of the intronic ectopic mutant splice acceptor site would restore normal *OTC* mRNA splicing and the production of normal protein and enzyme activity. Figure 1D summarizes the strategy for *ex vivo OTC* gene correction, hepatocyte transplantation to FRGN mice, and characterization of the resulting phenotype. To this end, combinations of paired guide RNAs (gRNAs) were tested for the efficient deletion of the ectopic splice site (Figure S1; Table S1) on induced pluripotent stem cells generated from the OTCD donor to conserve primary hepatocytes. One efficient pair, gRNA5 and gRNA8, was selected for experiments on primary hepatocytes. Primary OTCD hepatocytes were electroporated with ribonucleoproteins (RNPs) consisting of the synthetic gRNA pair and *Streptococcus pyogenes Cas9* (*Sp*Cas9) nuclease. Equal numbers of electroporated and un-electroporated cells were plated, and the results are shown in Figure 2A. Cas9-mediated editing efficiency was estimated to be on average >60% based on PCR amplicon analysis of the region of interest (Figures 2B and S2). Restoration of the normal splicing of the *OTC* gene was verified by RT-PCR and sequencing (Figures 2C and S3). Two different lengths of transcript were visualized from RT-PCR corresponding to mutant and residual wild-type mRNA, while a single band of wild-type size was observed for OTCP and edited OTCD hepatocytes (Figure 2C). The absence of mutant transcript in the corrected OTCD cells despite only partial correction of genomic DNA is likely due to the increased stability of wild type, corrected compared to mutant *OTC*, mRNA, which may be undergoing nonsense mediated decay (NMD).

With confirmation of the successful editing of the *OTC* locus based on genomic and transcriptomic analysis, restoration of functional urea cycle activity was next investigated. Primary OTCD hepatocytes were incubated in culture with $^{15}NH_4Cl$ to assess the incorporation of the stable isotope into newly synthesized urea. As shown in Figure 2D, the ratio of labeled to total urea produced in unedited (OTCD) cells was at background levels, while the edited, corrected OTCD hepatocytes incorporated significant amounts of ^{15}N from ammonia into urea, with a peak in activity on day 4 post-transfection, consistent with the restoration of OTC activity and urea cycle function. In addition, hepatocytes from 3 freshly isolated OTCP donors were investigated and the ratio of labeled to total urea was analyzed on day 4 of culture. The results differed between the donors; however, the gene-edited OTCD hepatocytes produced labeled urea at levels that were in the range that was observed in the OTCP hepatocytes (Figure 2E).

Genetically corrected human hepatocytes from the OTCD patient efficiently repopulate the FRGN mouse liver

The safety of CRISPR-based genome editing techniques involving double-stranded DNA break generation has been questioned in recent publications highlighting the risk of compromising genome integrity, and presumably affecting both the proliferation and function of genome-edited cells. To evaluate these factors, we compared the time required to reach 4 mg/mL serum human albumin between the groups of mice that received edited or unedited OTCD hepatocytes. This level of albumin corresponds to an estimated 80% repopulation of the mouse liver with human hepatocytes.[14,15] There was no significant difference between the two groups in the time it took to reach 4 mg/mL serum human albumin with genetically edited or unedited cells (Figure 3A). Levels of circulating human albumin over

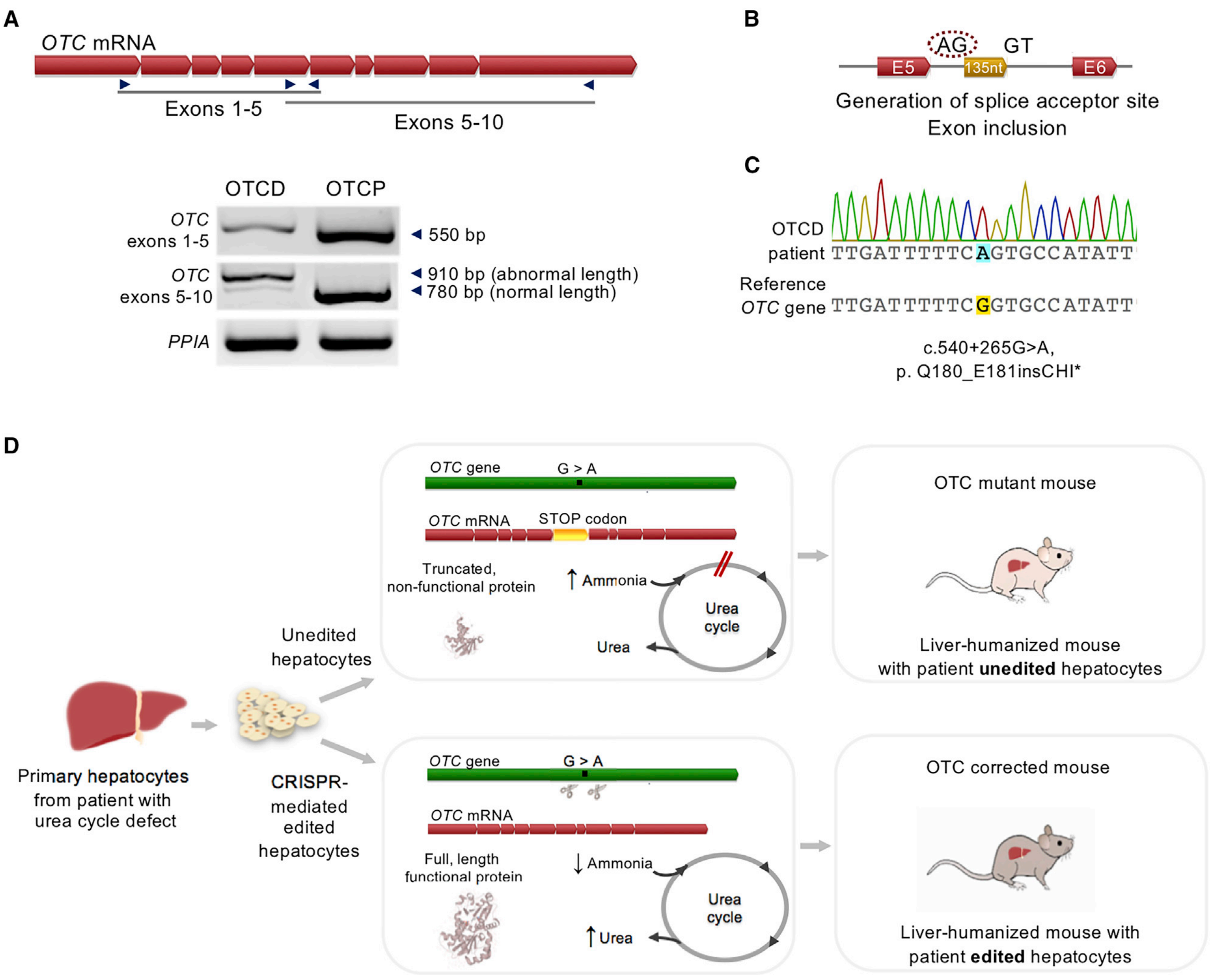

Figure 1. Disease-causing variant identification and study outline

(A) Amplification of *OTC* transcript in hepatocytes from OTC-deficient (OTCD) patient and OTC-proficient (OTCP) donor. Red arrow-shaped boxes represent exons of the *OTC* transcript, which were amplified with PCR primers indicated, spanning exons 1–5 and 5–10. The length of each amplicon product is indicated with arrows. Peptidylprolyl isomerase A (*PPIA*) transcript was used as an endogenous control. (B) Sequencing of patient *OTC* transcript revealed a 135-nt sequence inclusion, identical to an intronic region, between exons 5 and 6. Genomic structure of exons 5 and 6 of the *OTC* gene and the intronic region included in the transcript are shown. Red arrow-shaped boxes and horizontal lines represent exons and introns, respectively. Dinucleotides of the generated splice acceptor (AG) and the naturally existing donor (GT) flank the 135-nt insertion (yellow box). (C) Sequencing of *OTC* intronic region containing the mutation and alignment to reference gene (NCBI Gene: 5009). The genetic mutation (c.540+265 G > A) results in the generation of a splice acceptor (AG) site inserting an intronic region in the *OTC* transcript and generating a premature stop codon shortly after exon 5 (p. Q180_E181insCHI*). (D) Graphical depiction of the mutation identified, the consequences of the mutation, and the corrective strategy, followed by the characterization of the phenotype of the liver-humanized mice generated with unedited and edited cells.

time are presented in Figure S4, while those at the time of ammonia challenge are presented in Figure S5. In addition, the weights of animals transplanted with either unedited or edited OTCD cells were within the range that was observed when un-electroporated OTCP human hepatocytes were transplanted (Figure S6).

Different groups of mice were transplanted with "normal" hepatocytes isolated from livers known to be OTCP, unedited OTCD hepatocytes, or gene-edited OTCD hepatocytes. Highly repopulated chimeric mouse livers were generated from three separate *ex vivo* editing experiments with edited OTCD human hepatocytes. The efficiency of the successful editing of the OTCD hepatocytes and the repopulated livers was analyzed based on the quantification of the amplicon for the region of interest by densitometry (Figures 3B and S7). Editing efficiencies of the hepatocytes originally transplanted into the mice were evaluated along with liver tissues isolated from the highly repopulated animals from each transplant group. Experiments 1 and 2 show that ~80% of the human hepatocytes were successfully edited

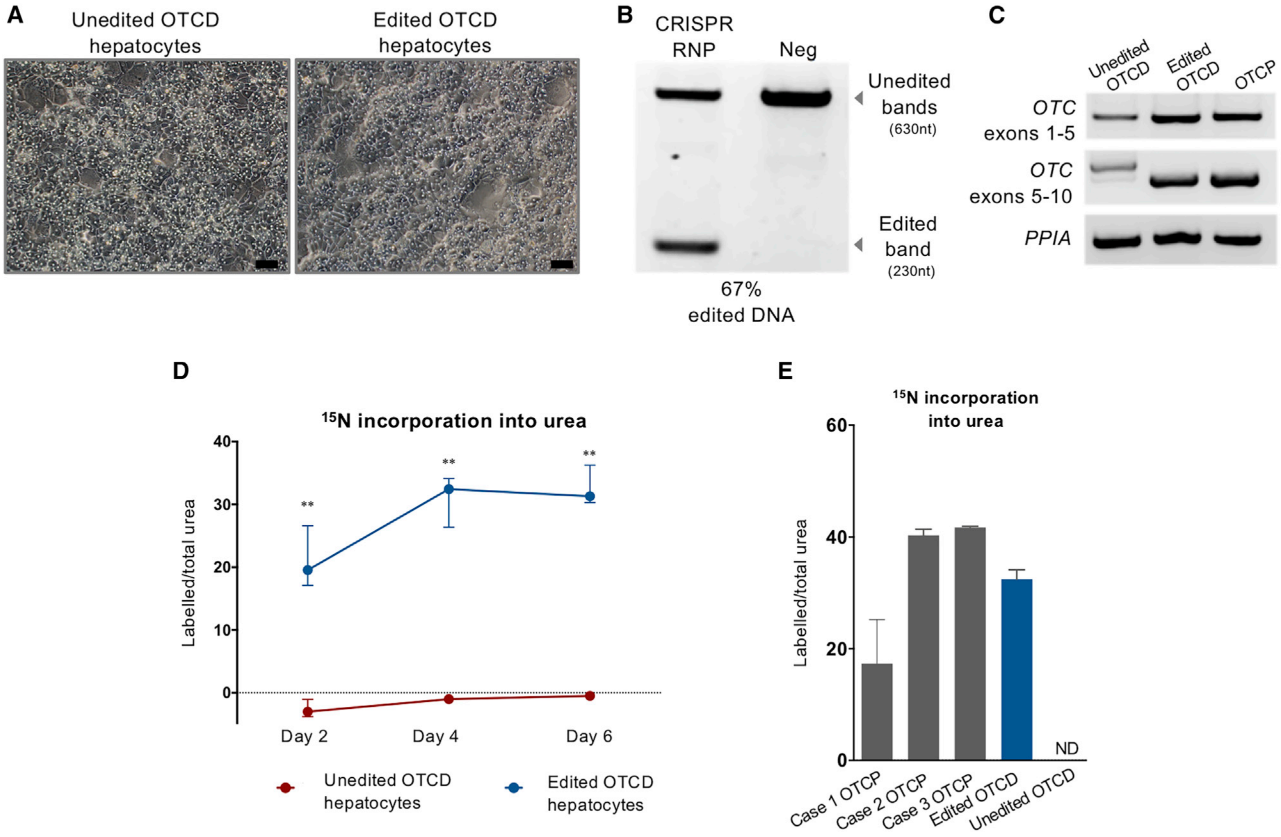

Figure 2. Genetic correction and *in vitro* phenotype characterization

(A) Phase contrast microscopy imaging of unedited or edited OTCD hepatocytes *in vitro* 2 days post-electroporation. Scale bar, 50 μm. (B) PCR amplification of targeting region in edited and unedited OTCD hepatocytes. Upper and lower bands indicate unedited (wild type) and edited DNA, respectively. Cleavage efficiency was estimated to be 67% based on band intensity and amplicon length. (C) PCR amplification of *OTC* transcript in unedited or edited OTCD hepatocytes, as well as OTCP cells. Transcripts of 2 lengths were observed in unedited OTCD hepatocytes, with a >100-nt difference, which corresponds to the intronic region included in the transcript. *PPIA* transcript was used as an endogenous control. (D) Phenotypic characterization *in vitro* assessed through ^{15}N incorporation into urea. Unedited and edited OTCD hepatocytes were incubated with $^{15}NH_4Cl$. Ratios of labeled to total urea produced by the cells on days 2, 4, and 6 post-electroporation are presented in the graph. Values and errors represent medians and interquartile ranges (unedited cells n = 6, edited cells n = 6 on each day). Statistical analysis was performed using the Mann-Whitney *U* test. Day 2, p = 0.0043; day 4, p = 0.0022; and day 6, p = 0.0022. (E) Phenotypic characterization *in vitro* assessed through ^{15}N incorporation into urea. Ratios of labeled to total urea produced by edited and unedited OTCD hepatocytes, as well as primary hepatocytes from 3 OTCP donors assessed on day 4 of culture, are shown. Values and errors represent medians and interquartile ranges (OTCP cases n = 3; edited and unedited OTCD cells n = 6). ND, not detectable.

in the cells originally transplanted into the mice. Liver tissue recovered from the highly repopulated animals showed the same level of successfully edited DNA. Editing of the hepatocytes in experiment 3 was less effective (~60%); however, the DNA from the highly repopulated animals in 2 of the 3 experiments was consistent with the editing efficiency of the hepatocytes originally transplanted, while the editing efficiency in experiment 3 was lower. However, all of the experiments demonstrate that Cas9 nuclease-edited hepatocytes repopulate the FRGN liver as efficiently as the unedited cells.

Immunohistochemical analysis was conducted on serial sections of liver with antibodies specific for human cytokeratin (CK) 8 and 18 or human OTC from all 3 experimental groups. All human hepatocytes in the mouse liver should react with antibodies to CK. However, the disease-causing variant in the OTCD cells introduces a premature stop codon shortly after exon 5, such that the antibody does not recognize residual mutant OTC protein in the hepatocytes. As shown in Figures 3C and S8, in mice that received unedited OTCD hepatocytes, numerous CK^+ colonies of human hepatocytes are detected; however, there is virtually no staining for OTC protein. Mice transplanted with corrected, gene-edited OTCD hepatocytes display high-level repopulation of the liver with hepatocytes, as demonstrated by CK staining on >80% of the liver. Moreover, the analysis with the anti-OTC antibody reveals a chimeric liver consisting of large colonies of OTC^+ human hepatocytes, presumably originating from OTC-corrected human hepatocytes interspersed with equally large colonies of cells that still react with antibodies to human hepatocyte cytokeratins, but that are not detected with antibodies for human OTC, which presumably originate from the unedited, original OTCD hepatocytes. In summary, >60% of the human cells in the liver section from mice that were transplanted

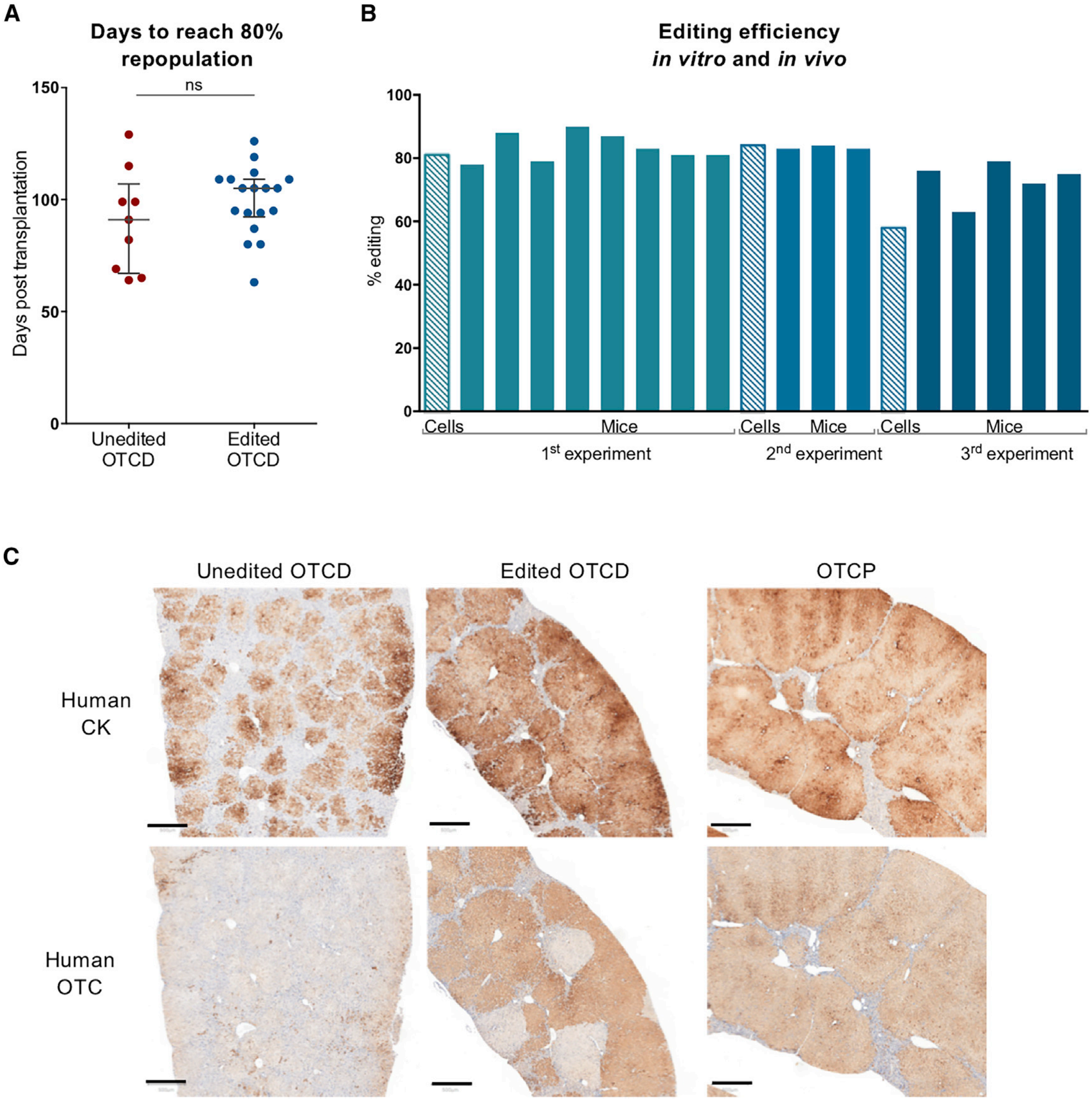

Figure 3. Editing efficiency and immunohistochemical analysis of humanized mouse livers

(A) Comparison of repopulation capacity between unedited and edited OTCD hepatocytes in FRGN mice. Days required for the cells to repopulate mouse liver to an estimated level of ≥80% (≥4 mg/mL circulating blood human albumin). Unedited OTCD mice n = 9. Edited OTCD mice n = 18. Statistical analysis was performed using 2-tailed, unpaired t test, p = 0.2904. (B) Estimation of editing efficiency in hepatocytes before transplantation (striped bars) and in liver-humanized mice with the respective cells (full-color bars). Three independent transplantation experiments were carried out. Each bar represents editing efficiency either *in vitro* or in a humanized mouse liver with the respective cells. (C) Representative sections of mouse liver immunostained with antibodies against human CK or OTC in mice that received either unedited or edited OTCD or OTCP human hepatocytes. Areas positive for CK indicate the areas repopulated with human hepatocytes. Positive reaction with antibodies to OTC indicates cells that express full-length, normal protein. Scale bar, 500 μm.

with edited cells now react with antibodies to OTC, which is consistent with the expected editing efficiency of the OTCD hepatocytes originally transplanted into mice.

Normal basal ammonia metabolism is restored in mice repopulated with gene-edited OTCD hepatocytes

The standard of care for severely affected OTCD patients is a protein-restricted diet, usually supplemented with ammonia scavengers to prevent hyperammonemic episodes.[4] Throughout these studies, all of the mice were maintained on a non-restricted, regular (19%) protein diet without the administration of ammonia scavengers. Basal blood ammonia levels in mice transplanted with unedited OTCD hepatocytes were significantly higher than those transplanted with edited OTCD cells (median 206.9 and 65.1 μM, respectively, p = 0.0018). Mice that were repopulated with corrected, gene-edited OTCD hepatocytes displayed ammonia levels not significantly different from control mice transplanted and highly repopulated with normal OTCP human hepatocytes (median 65.1 and 59.4 μM, respectively, p >

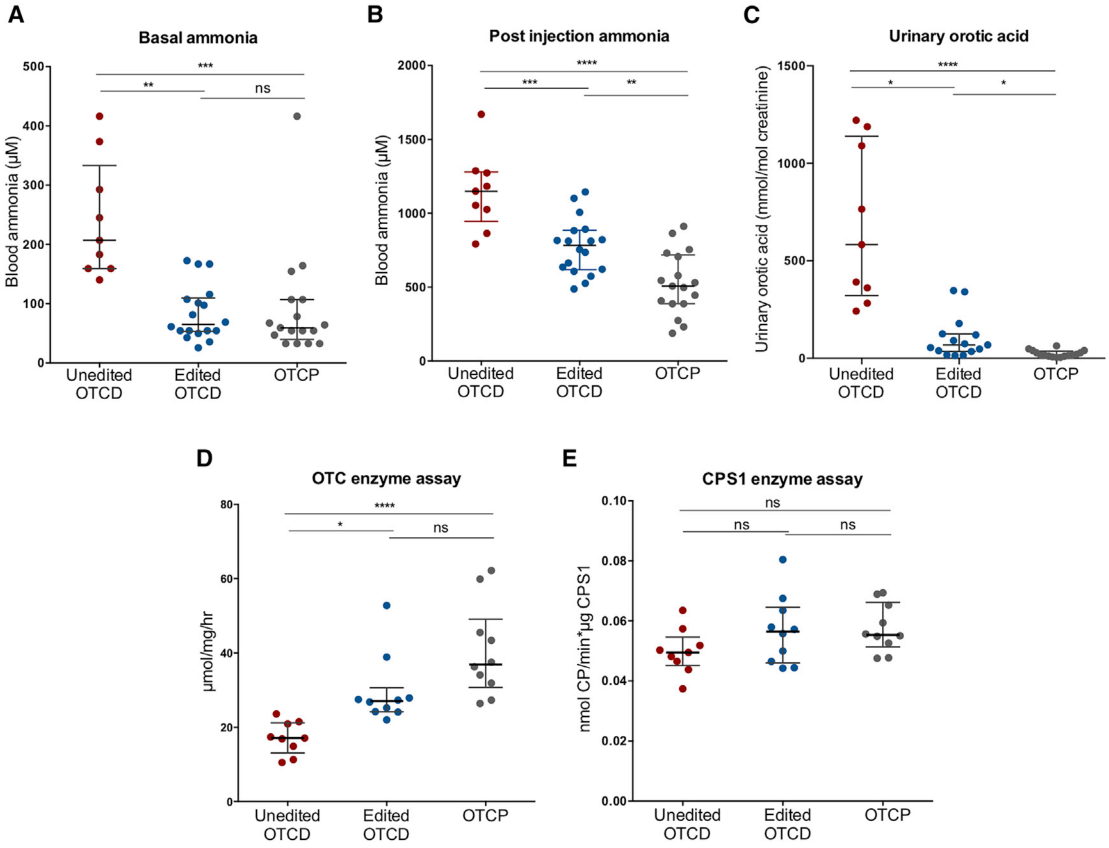

Figure 4. *In vivo* phenotype characterization of mice repopulated with unedited and edited OTCD or OTCP hepatocytes

(A) Basal blood ammonia levels in humanized mice. Unedited OTCD mice n = 9. Edited OTCD mice n = 18. OTCP mice n = 17. Unedited OTCD versus edited OTCD, p = 0.0018; unedited OTCD versus OTCP, p = 0.0004; edited OTCD versus OTCP, p > 0.9999. (B) Blood ammonia levels in humanized mice measured 30 min post-injection of $^{15}NH_4Cl$ (ammonia challenge). Unedited OTCD mice n = 9. Edited OTCD mice n = 18. OTCP mice n = 17. Unedited OTCD versus edited OTCD, p = 0.0003; unedited OTCD versus OTCP, p < 0.0001; edited OTCD versus OTCP, p = 0.0041. (C) Urinary orotic acid in humanized mice normalized to urinary creatinine. Unedited OTCD mice n = 9. Edited OTCD mice n = 15. OTCP mice n = 16. Unedited OTCD versus edited OTCD, p = 0.0158; unedited OTCD versus OTCP, p < 0.0001; edited OTCD versus OTCP, p = 0.0268. (D) OTC enzyme activity in humanized mouse livers. Unedited OTCD mice n = 9. Edited OTCD mice n = 10. OTCP mice n = 10. Unedited OTCD versus edited OTCD, p = 0.0112; unedited OTCD versus OTCP, p < 0.0001; edited OTCD versus OTCP, p = 0.3452. (E) CPS1 enzyme activity in humanized mouse livers. Unedited OTCD mice n = 9. Edited OTCD mice n = 10. OTCP n = 10. Unedited OTCD versus edited OTCD, p = 0.2486; unedited OTCD versus OTCP, p = 0.1743; edited OTCD versus OTCP, p = 0.9745. Averages and errors are shown as medians and interquartile ranges. Kruskal-Wallis ANOVA and Dunn's multiple comparison tests were used to perform statistical analysis for datasets not normally distributed (basal ammonia, urinary orotic acid, and OTC enzyme assay), while ordinary ANOVA and Tukey multiple comparison tests for datasets normally distributed (post-injection ammonia and CPS1 enzyme activity).

0.9999) (Figure 4A). All of the human hepatocyte-repopulated mice were subjected to an ammonia challenge by injection of NH_4Cl (4 mmol/kg body weight) and blood ammonia was measured 30 min post-administration. The post-injection ammonia levels were significantly different between mice transplanted with unedited and edited OTCD hepatocytes (median 1,149 and 782.8 µM, respectively, p = 0.0003). However, the ammonia levels in the gene-edited OTCD group were still higher than those of mice transplanted with OTCP human hepatocytes (782.8 and 506.7 µM, respectively, p = 0.0041), consistent with the 60%–80% genetic editing and partial correction of the phenotype (Figure 4B).

In addition to hyperammonemia, the presence of orotic acid in the urine is a characteristic surrogate marker of OTCD because accumulated carbamoyl phosphate, a substrate for OTC, enters the pyrimidine nucleotide synthesis pathway, which produces orotic acid. Urinary orotic acid in mice that received gene-edited OTCD hepatocytes displayed vastly improved urinary orotic acid levels compared to those that received unedited OTCD (Figure 4C), and nearly half of the mice displayed orotic acid levels in the range of those found in the OTCP group.

Measurements of OTC enzyme activity in the livers of mice repopulated with edited hepatocytes were significantly higher than the levels in mice transplanted with unedited cells (median 27.1 and 17.1 µmol/mg/h, respectively, p = 0.0112), and these were not significantly different from the levels observed in mice transplanted with OTCP hepatocytes (median 27.1 and 36.9 µmol/mg/h, respectively, p = 0.3452) (Figure 4D).

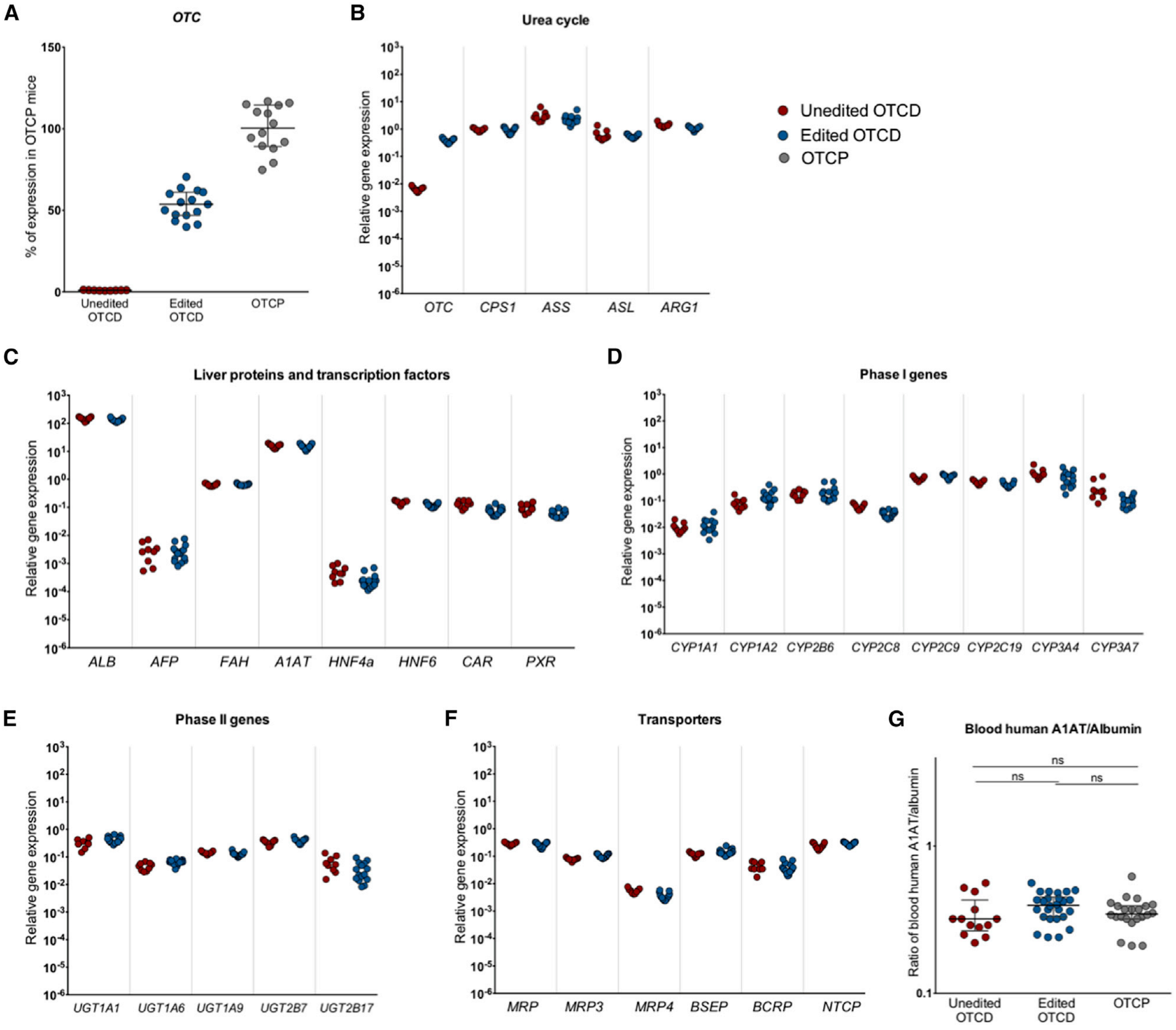

Figure 5. Gene expression and blood human markers

(A) *OTC* gene expression in livers humanized with unedited (red) or edited (blue) OTCD cells presented as percentage of mean OTC expression in humanized livers with OTCP cells (gray). Averages and errors are shown as medians and interquartile ranges. Unedited OTCD mice n = 9. Edited OTCD mice n = 15. OTCP mice n = 15. (B–F) Gene expression analysis of humanized livers that received either unedited (red) or edited (blue) OTCD cells. Expression of mature liver genes was assessed, including urea cycle genes (B), liver-secreted proteins and transcription factors (C), phase I (D), and phase II (E) genes, as well as transporters (F). Unedited OTCD mice n = 9. Edited OTCD mice n = 15. (G) Ratio of circulating human alpha-1-antitrypsin (A1AT) to albumin were measured in the blood of mice repopulated with unedited (red) or edited (blue) OTCD cells or OTCP (gray) cells. Unedited OTCD n = 13 measurements from 6 mice. Edited OTCD n = 26 measurements from 14 mice, OTCP n = 18 measurements from 11 mice at different levels of repopulation. Data are shown as medians and interquartile ranges. Statistical analysis was performed using Kruskal-Wallis ANOVA and Dunn's multiple comparison tests. Unedited OTCD versus edited OTCD, p = 0.1405; unedited OTCD versus OTCP, p > 0.9999; edited OTCD versus OTCP, p = 0.4967.

The activity of another urea cycle enzyme, CPS1, was not significantly different between any of the transplant groups (Figure 4E).

Hepatocyte editing does not disrupt normal gene expression in *ex vivo* corrected hepatocytes and shows no evidence of off-target editing

Having established an efficient *OTC* gene correction, the next step was to evaluate the safety of *OTC* editing for a potential CRISPR-based therapy. One concern for CRISPR-mediated gene editing is the unintended and undesirable effects of on- or off-target editing, which could alter normal gene expression and cell/organ function. To address this, gene expression profiling was conducted to examine the expression of genes in the liver of mice humanized with OTCD edited, OTCD unedited, and OTCP cells. *OTC* and an additional set of 31 genes, including those from the urea cycle, secreted proteins, transcription factors, phase I and phase II, hepatic transport proteins, and other metabolic

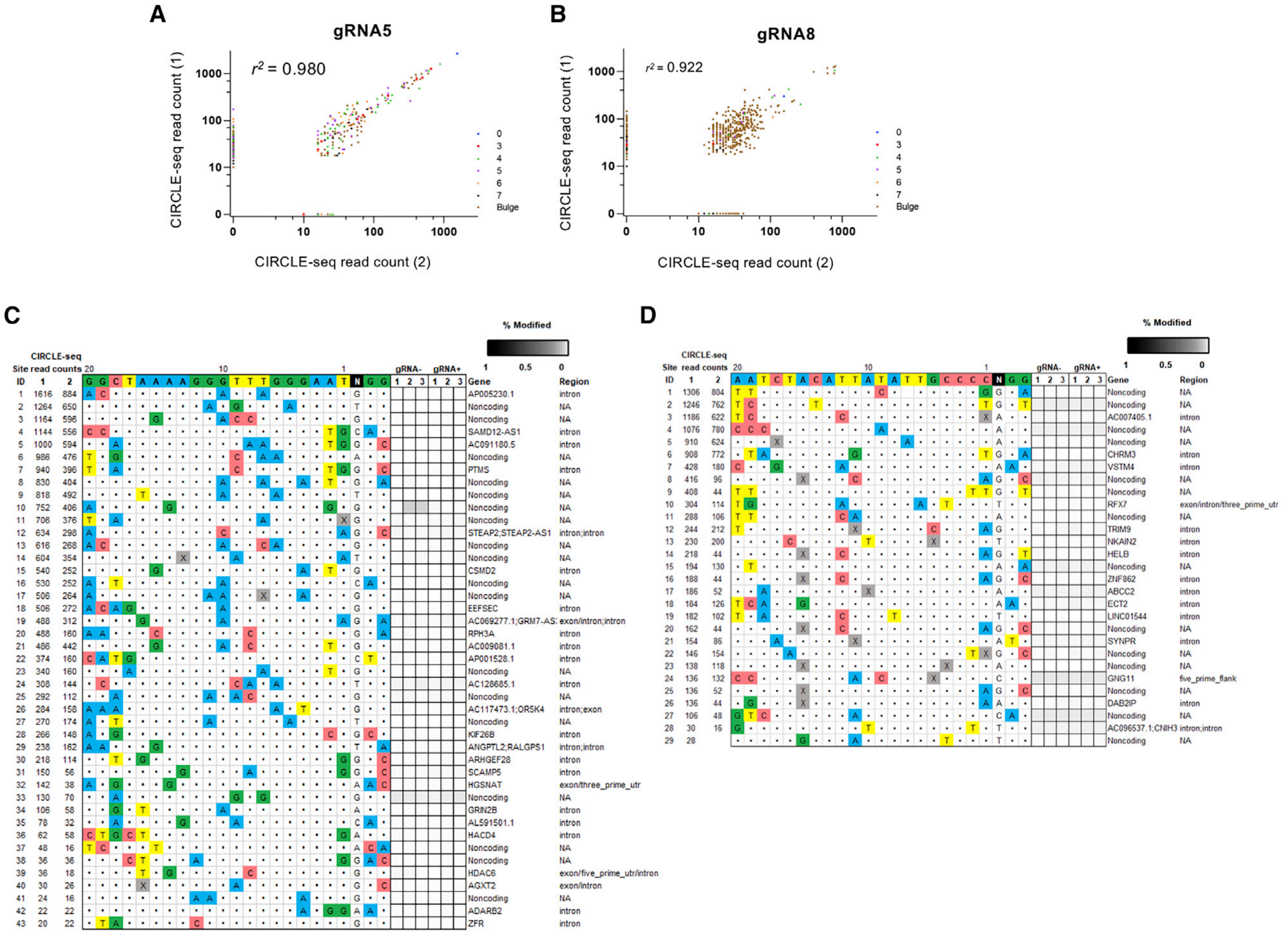

Figure 6. Off-target editing

(A and B) Off-target discovery of OTC-targeting gRNAs by CIRCLE-seq. Scatterplots of CIRCLE-seq read counts for off-target cleavage sites identified *in vitro* with Cas9-gRNA5 (A) or Cas9-gRNA8 (B) in 2 replicates on genomic DNA from patient-derived primary hepatocytes. Read counts are shown on a logarithmic scale. Each site is color coded for the number of mismatches relative to the on-target site. Correlation r^2 values in between 2 replicates were obtained by Pearson correlation performed using all values shown in the scatterplot. (C and D) Assessment of off-target indels induced by Cas9-gRNA5 (C) or Cas9-gRNA8 (D) in patient-derived primary hepatocytes. Indel frequencies as determined by targeted amplicon sequencing are presented as heatmaps for the off-target sites (identified from CIRCLE-seq experiments). CIRCLE-seq read-count numbers for each site are listed. Mismatches relative to the on-target site are shown with colored boxes, whereas bulges are indicated with X. Each locus was assayed in n = 3 transfection replicates (labeled 1, 2, and 3) using genomic DNA isolated from the cells electroporated with RNP complex Cas9-gRNA (gRNA+) or control-only Cas9 protein (gRNA−). Genomic identifications and locations for each locus are presented. NA, not applicable.

pathways, were analyzed by qPCR (Figures 5A–5F). The assays used were human specific to avoid cross-reactivity with any remaining mouse cells in the liver. Mice transplanted with unedited OTCD cells showed only 0.1% of the expression of the OTC gene in comparison to OTCP cells, whereas transplantation of edited OTCD cells restored OTC gene expression to 55% of that observed in normal OTCP cells (Figure 5A). As shown in Figures 5B–5F, the expression of the other 31 mature liver genes was very similar between the unedited and edited hepatocytes in the liver of repopulated mice. Human alpha-1 antitrypsin (A1AT), a glycoprotein secreted by the hepatocytes, was also measured in the mouse sera from the three experimental groups. Because there are small variations in the level of repopulation of the liver of different mice, circulating A1AT values were normalized to the levels of another secreted protein, human albumin, and there was no statistically significant difference in the normalized A1AT levels between the groups (Figure 5G).

To assess genome-wide off-target mutations induced by Cas9-gRNA5 and Cas9-gRNA8, we conducted a genome-wide off-target screening by performing circularization for *in vitro* reporting of cleavage effects by sequencing (CIRCLE-seq), with Cas9 on genomic DNA isolated from unedited primary hepatocytes.[18] These experiments identified a number of *in vitro* off-target cleavage sites: for gRNA5 167 off-targets common in 2 CIRCLE-seq replicates (Figure 6A; Table S3); and for gRNA8, 421 off-targets common in both replicates (Figure 6B; Table S4). The sites that were identified in both replicates showed

strong concordance in their CIRCLE-seq read counts between the 2 replicates (r^2 = 0.980 and 0.922, for gRNA5 and gRNA8, respectively) (Figures 6A and 6B). Sites that are identified in only one replicate generally had low CIRCLE-seq read counts (Figures 6A and 6B). Among the off-targets common in both replicates, none of them showed fewer than three mismatches compared to on-target sites.

We next performed targeted amplicon sequencing of potential gRNA5 and gRNA8 off-target sites found by CIRCLE-seq, using genomic DNA isolated from three replicates of edited OTCD primary hepatocytes and their respective Cas9 only-treated controls. We assessed all off-targets that were common in both CIRCLE-seq replicates with up to three mismatches relative to the on-target site, or with read counts >10% of the top ranked target in both replicates; and a variety of lower-ranked sites (containing up to 6 mismatches relative to the on-target) (Figures 6C and 6D). The selection was based on our previous findings showing that the likelihood of the *in vivo* off-target mutagenesis is dependent on the number of mismatches and read counts of the off-targets identified by CIRCLE-seq, and all validated *in vivo* off-targets had up to three mismatches.[19] One site for gRNA5 and two sites for gRNA8 could not be successfully amplified and therefore were not included in amplicon sequencing. Among the evaluated 43 off-target sites for gRNA5 and 29 off-target sites for gRNA8, we found no significant indel mutations at any of the sites (Figures 6C and 6D; Tables S5 and S6) within the next-generation sequencing (NGS) limit of detection. These results further confirm the conclusion of our previous work showing that the off-target effect of CRISPR/Cas9 is limited in differentiated cells with carefully designed guides.

DISCUSSION

The present study was performed to determine whether (1) liver-humanized mice, created through the high-level repopulation of the liver with human hepatocytes isolated from a patient with severe OTCD, would recreate the phenotype and symptoms of the human genetic disease, and (2) CRISPR-based *ex vivo* gene editing procedures could correct the mutation and the disease phenotype without causing any deleterious effects or undesired mutagenesis. The present work demonstrates a useful chimeric model system to investigate the safety and efficacy of gene editing technology for OTCD and possibly other genetic liver diseases. When OTCD patient-derived human hepatocytes were transplanted, they rapidly and efficiently repopulated the FRGN mouse liver. Repopulated mice replicated the human OTCD clinical phenotype, presenting elevated blood ammonia and increased urinary orotic acid. The intronic OTCD mutation that leads to truncated protein generation was corrected in patient hepatocytes by *ex vivo* Cas9 nuclease-mediated editing, which substantially restored normal mRNA splicing and OTC activity. When FRGN mice were transplanted with highly edited (60%–80%) or unedited hepatocytes, both repopulated the mouse liver with similar efficiencies, indicating that the editing step was well tolerated by the liver cells and did not seriously adversely affect the proliferative potential of the edited cells. In mice that received edited hepatocytes, disease indications were largely corrected as the animals displayed normal blood ammonia, highly improved urinary orotic acid levels, and OTC enzymatic activity in the liver that was not significantly different from OTCP human hepatocytes. This level of correction of the OTCD phenotype is notable because all of the mice were maintained on a regular protein diet throughout the experiment and no animals received ammonia scavengers. The deletion of the region containing the disease-causing variant in the *OTC* gene efficiently reversed the OTCD phenotype *in vitro* and *in vivo*, and editing procedures significantly improved OTC enzyme activity, with no other obvious morphological or pathological changes or major alterations in the expression of the 31 mature liver genes examined. Surprisingly, while basal ammonia was completely normalized in the edited group, the same group could clear ammonia faster when subjected to ammonia challenge, but not at the same magnitude as healthy controls. This may be attributed to the partial correction, and perhaps higher efficiency of gene editing may be required. Given that complete urea cycle activity and ammonia metabolism are tightly regulated and restricted to zone 1 hepatocytes (i.e., those nearest the portal veins), a disruption or failure to completely reproduce the normal zonation of the liver in liver-humanized mice would be expected to influence ammonia metabolism. This may help explain how animals repopulated with hepatocytes with an approximate level of correction of 80% resulted in the normalization of the basal ammonia levels, but still revealed a significant although delayed clearance of ammonia during the ammonia challenge.

Hepatocytes were one of the first cell types to be shown to be amenable to CRISPR-based genome editing *in vivo* in rodents for both non-homologous end joining (NHEJ) or homologous-directed repair (HDR). The elegant studies of Yang et al.[12] described the use of *in vivo* dual-adeno-associated virus (AAV)-delivered CRISPR gene editing components to repair the OTC gene in the sparse fur ash (*spf*^{ash}) rodent model of OTC deficiency. An impressive 10% of hepatocytes were precisely repaired in neonatal animals by the combination of a directed Cas9 double-stranded DNA break and a DNA repair template for HDR. However, when the same reagents were delivered into adult mice, lower levels of precise gene correction were observed along with the generation of unintended genome damage that inactivated residual OTC activity and compromised the health of the edited animals. These studies in a murine OTCD model highlight both the promise and unpredictable safety risks of CRISPR nuclease-mediated therapeutic genome editing. Human hepatocytes are absolutely necessary in preclinical investigations, as we have shown here and reported by others.[20]

Several observations were made during the studies that are relevant to possible future clinical applications of CRISPR gene editing to treat liver-based genetic diseases. Gene editing of human hepatocytes can be accomplished with a high degree of efficiency and specificity, with 60%–80% successful editing in these studies. We note that we are using CIRCLE-seq as an explorative tool to address the level of off-target in differentiated cells, since most of the off-target validation studies have been performed in immortalized cell lines. CIRCLE-seq generates a superset of off-targets overestimating the real scenario in

cells or *in vivo*, because the *in vitro* reaction does not depend on the delivery success of Cas9/gRNA to the cells since the method uses high amounts of Cas9/gRNA and naked genomic DNA does not have secondary structures such as chromatin. We have therefore explored the worst-case scenario and we include in our analysis CIRCLE-seq-identified off-targets with up to three nucleotide substitutions and off-targets showing the highest number of read counts regardless of mismatches (top 10% and an additional variety of lower-ranked sites). The selection is based on our previous findings that reported that the likelihood of the *in vivo* off-target mutagenesis is dependent on the number of mismatches and read counts of the off-targets identified by CIRCLE-seq.[19] Our results showed no CIRCLE-seq-identified off-targets with more than three mismatches were found mutated *in vivo*. We therefore do not expect to detect off-targets in the remaining unvalidated sites within the current NGS limit of detection (0.1%), as discussed in our previous work. Here, we used CIRCLE-seq to evaluate the safety of gRNAs that are designed to correct a specific OTC mutation; however, the method can be easily tailored for gRNAs designed to correct any genomic target, including other OTC mutations. None of the sites were shown to be unintentionally altered, emphasizing that the combination of well-selected CRISPR target sites and transient RNP-based delivery can achieve high on-target editing efficacy, while causing no detectable off-target effects.

Gene-edited human hepatocytes maintain the ability to robustly proliferate and can contribute proportionately with unedited cells in the repopulation of the liver of the FRGN mice. The level of editing measured in the human hepatocytes originally transplanted into mice was maintained in the highly repopulated and humanized chimeric liver, indicating that there was no major selection either for or against the gene-edited cells during the repopulation process. Additional evidence that gene editing did not adversely affect hepatocyte proliferation or survival was provided in that mice that received edited or unedited hepatocytes reached similar levels of repopulation with human hepatocytes (4 mg/mL) over the same time period. It is worth mentioning that mice were subjected to ammonia assays and harvested for sequencing and other evaluations at different humanization levels. Specifically, the unedited OTCD group was terminated when it reached at least 4 mg/mL, which corresponds to >80% humanization, and the edited OTCD and OTCP groups reach at minimum 5 mg/mL, which corresponds to full humanization. The reason for setting a different threshold was to prevent the most severe symptoms of OTCD, including hyperammonemia and weight loss, as the ~20% of remaining mouse hepatocytes in the unedited group would sustain some basal levels of ammonia metabolism. Despite retaining up to 20% OTCP mouse hepatocytes in the unedited animals, there were significant differences in ammonia levels between unedited and edited groups. Forcing an experiment by trying to follow animals to a higher level of repopulation with OTCD cells would likely only magnify the differences between the unedited and edited groups, but at a price to animal health and well-being, which was biologically and statistically unnecessary but also ethically untenable. Still, it is recognized that a potential limitation of the present study is that animals were analyzed at different levels of repopulation.

While the data presented here indicate that gene editing is safe and effective in human hepatocytes, certain questions remain. The mouse model used in this study is severely immunocompromised and the question of whether CRISPR components could trigger an immune reaction cannot be addressed in these animals. We believe that other studies with immunocompetent animals will be more relevant to the topic of immunogenicity of editors than anything conjured with the liver-humanized mice.

A potential limitation of the study is the correction of the disease mutation through the NHEJ repair pathway; however, with >400 reported mutations affecting OTC activity, every approach to correct mutations in these severely affected patients is valuable.

We did not specifically investigate the possible presence of large rearrangements caused by the editing process by deep sequencing. Recent reports suggest that large deletions were infrequent during repair processes.[21,22] Also, if such events occurred, they did not have significant or long-term biological consequences in these studies. The restoration of *OTC* expression and activity correlated well with our reported editing efficiencies, and the observations that the edited and unedited cells displayed robust rates of proliferation and expression of >30 mature liver genes suggest that any deleterious editing events were selected against or remained greatly underexpressed in the population of cells that repopulated and formed the fully functional liver. In the present study, the clonal growth of large colonies of human hepatocytes, presumably derived from single, edited, or unedited hepatocytes, was observed. Generation of a colony of 250–1,000 cells from a single cell would require a minimum of 8–10 population doublings. Despite the massive proliferation of the edited hepatocytes in this study, no pathological changes or other obvious safety issues were noted.

Perhaps the most important development in this report is the establishment of a liver-humanized model in which the safety and efficacy of the editing procedures can be investigated with actual mutant human hepatocytes *in vivo*, where long-term studies can be conducted. Preclinical proof of concept work in therapeutic genome editing has traditionally been done with rodent models of genetic disease. However, the spectrum of on- and off-target effects of genomic CRISPR medicine will depend on the target, the human genomic sequences, and human-specific repair pathways. Thus, the extrapolation of the data from rodent studies to predict a response in humans is likely of only limited value. For hepatocyte-based diseases, there is a clear advantage for performing preclinical editing studies with human hepatocytes and with actual disease-affected human hepatocytes when possible. Chimeric transplantation systems such as the FRGN mouse enable the assessment of the ability of edited cells to regenerate a functional organ and can help generate valuable safety and efficacy data to support the evaluation of therapeutic genome editing.

The present study tests the biological consequences of the editing procedure and, given the highly selective and proliferative animal model used, it is not designed to determine whether *ex vivo* editing could correct the disease in an OTCD recipient. There are no data to suggest that edited and OTCP cells would have a growth advantage in an OTCD recipient; thus, it is unlikely that *ex vivo* editing and subsequent hepatocyte transplantation would be a successful strategy. For successful translation to the clinic, *ex vivo* editing of hepatocytes would be most beneficial in diseases in which the corrected hepatocytes would be expected to have a growth advantage over the remaining uncorrected, diseased cells in the liver. Diseases such as A1AT deficiency, the family of bile acid pathway disorders called progressive familial intrahepatic cholestasis (PFIC 1, 2, or 3), or Wilson's disease are examples in which there is continued toxicity or impaired replication of the disease-affected cells. With these diseases, one could expect a significant growth advantage for gene-edited and corrected hepatocytes.[23–25]

Previous work involving successful *in vivo* CRISPR editing of human hepatocytes in chimeric FRGN mice targeting the *PCSK9* locus with adenoviral vectors demonstrated the strengths of using this model system for efficacy studies of therapeutic genome in an *in vivo* context; however, the adenoviral approach would not be likely translated to human use.[26]

In summary, this study provides evidence that gene editing of hepatocytes isolated from a patient with a serious genetic disease safely and efficiently corrects the disease phenotype and restores liver function. Moreover, the work demonstrates the value of the liver-humanized models for the evaluation of the efficacy and long-term safety of novel therapies for clinical translation.

MATERIALS AND METHODS

Primary hepatocytes

Human primary hepatocytes were procured at Karolinska Institutet under ethical approval Dnr2010/678-31/3. Hepatocytes were obtained from an 8-month-old male donor who received a liver transplant for severe OTCD. Normal OTCP human hepatocytes were obtained from an 8-year-old male, a 35-year-old female organ donor, and 2 tissue donors who received liver resections for colorectal cancer metastasis—a 77-year-old male and a 27-year-old male. Hepatocytes from the OTCD and the 8-year-old male donor were used for transplant studies and were thawed on the day of transplantation. The viability of OTCD and OTCP hepatocytes used for transplant studies was 63% ± 0.06% and 83% ± 0.01%, respectively, and the viability of fresh cell donors was 85%, 74%, and 88%, respectively, according to Trypan Blue exclusion assays.

Generation of induced pluripotent stem cells (iPSCs)

Gene editing optimization experiments were performed on iPSCs generated from fibroblasts isolated from the liver from the OTCD patient. Cell reprogramming was conducted with Sendai virus-mediated forced expression of Yamanaka factors using the CytoTune-iPS Sendai Reprogramming Kit (Thermo Fisher) and following the manufacturer's instructions. iPSC lines obtained were characterized based on standard evaluation assays and gene expression.[27]

Mutation identification

Total RNA was isolated from OTCD hepatocytes with the PureLink RNA extraction kit (Life Technologies). Complementary DNA (cDNA) was synthesized with PrimeScript Reverse Transcriptase (Takara) according to the manufacturer's instructions. *OTC* transcripts were amplified with Pfu Ultra II Fusion HS DNA polymerase (Agilent Technologies) from exon 1 to exon 5 with forward primer 5′-GAAGATGCTGTTTAATCTGAGG and reverse primer 5′-CTGGAGC GTGAGGTAATCAGCC, and from exon 5 to exon 10 with forward primer 5′-GCAGATGCAGTATTGGCTCG and reverse primer 5′-CCCATACCACGTGTTAGGGATT, as described previously.[28] The suspected region containing the disease-causing *OTC* variant was amplified with forward primer 5′-TCTCATCCTCATGTCCAAA GTGTT and reverse primer 5′-TATGTAAAGCCACACCCACAGAC with Taq DNA Polymerase (NEB), and Sanger sequenced. Geneious version 8.1.9 was used for primer design, sequence view, alignment, and illustration throughout the study.

CRISPR design and transfection

The intronic region in the *OTC* gene containing the mutation was deleted using a dual synthetic single gRNA RNP approach. gRNAs were designed with the CRISPR Design Tool (https://crispr.mit.edu). Several gRNAs upstream and downstream of the disease-causing variant were chosen to be tested. The sequence and position of each gRNA in the gene are provided in Table S1. Cells were transfected with a P3 Primary Cell 4D-Nucleofector kit (Lonza), program CA137, with a Lonza 4D Nucleofector according to the manufacturer's instructions. Specifically, crRNA:tracrRNA was annealed to a final duplex concentration of 100 μM at 95°C for 5 min. Next, 1.8 μL of each gRNA of the pair to be transfected, 5 μL 61 μM *Streptococcus pyogenes* Cas9 (*Sp*Cas9), and 3 μL 100 μM electroporation enhancer were used for each reaction to transfect 10^6 cells. All crRNAs, tracrRNA, *Sp*Cas9 nuclease, and electroporation enhancer were purchased from Integrated DNA Technologies (IDT). Cells were maintained at +4°C until transplanted. Aliquots of transfection reactions were plated *in vitro* and analyzed for editing efficiency 24–48 h post-electroporation.

Genomic DNA extraction and editing quantification

Genomic DNA from iPSCs, hepatocytes, or humanized mouse liver tissues was extracted using DNeasy Blood and Tissue Kit (QIAGEN). The region of interest was amplified with forward primer 5′-TCTCATCCTCATGTCCAAAGTGTT and reverse primer 5′-TATGTAAAGCCACACCCACAGAC using Taq polymerase (NEB). PCR primers were human specific, eliminating cross-reactivity concerns when DNA from humanized mouse liver, which contains both human and mouse cells, was amplified. Cleavage efficiencies were estimated based on the intensities of unedited and edited bands of the PCR amplicons on 1.5% agarose gel. Intensities were corrected for the amplicon length of the reaction product and calculated as follows: Editing efficiency % = [(edited band intensity/

edited band length)/(unedited band intensity/unedited band length + edited band intensity/edited band length)]*100. The unedited band length was ≈630 nt. The edited band length from gRNAs 4–6 was ≈427 nt, gRNAs 4–7 ≈322 nt, gRNAs 4–8 ≈240 nt, gRNAs 4–9 ≈207 nt, gRNAs 5–6 ≈421 nt, gRNAs 5–7 ≈316 nt, gRNAs 5–8 ≈234 nt, and gRNAs 5–9 ≈201 nt. In addition, Agilent's Fragment Analyzer system was used as a quantitative measure of band intensities normalized to fragment size. For this analysis, the region of interest was amplified with forward primer 5′-ACCAGCTT-GACAAAGAATTACAGC and reverse primer 5′- GGGCCTATC-CAGTTTATCTTGTCT using Q5 High-Fidelity DNA polymerase (NEB).

In vitro ^{15}N-labeled urea production

Genetically edited or unedited hepatocytes were plated on collagen-coated plates and cultured in Hepatocyte Maintenance Medium (HMM, Lonza) media for analysis. Post-electroporation, cells were incubated in HMM with 1 mM ornithine (Sigma-Aldrich) and 1 mM $^{15}NH_4Cl$ (Sigma-Aldrich) for 4 h. Unlabeled (mass to charge [*m/z*] 231) and labeled urea (*m/z* 232 and 233) were measured with mass spectrometry (MS).[29]

For the measurement, media samples were deproteinized with cold aceton (VWR), centrifuged, and cleaned on 1 mL AG-50W-X8 resin (analytical grade 100–200 mesh, hydrogen form, Bio-Rad). After overnight drying in a rotary evaporator (Speedvac, Thermo Fisher), the samples were derivatized with ethyl acetate (VWR) and MTBSTFA (Biotech AB) (1/1) at 60°C for 60 min. The samples were analyzed for *m/z* on an Agilent GC-MS System (Gas Chromatograph 6890, Mass Spectrometer 5975) with a non-polar gas chromatography (GC) column (Agilent HP-5MS). *m/z* 231, 232, 233 were detected in electron ionization mode.

The amount of labeled urea (^{15}N-urea) was expressed as MPE (molar percent excess). MPE is calculated from the ratio of labeled:unlabeled urea (Rs) corrected for natural abundance of standard urea (R0): (Rs-R0)/(1+(Rs-R0)) × 100.

Hepatocyte transplantation

All of the animal studies were conducted according to Karolinska Institutet guidelines and under ethical protocol ID400 42-17 approved by ethics board Jordbruksverket, Sweden. FRGN (*Fah*$^{-/-}$, *Rag2*$^{-/-}$, and *Il2rg*$^{-/-}$ on the NOD-strain background) mice, approximately 6 weeks old at the time of transplantation, both females and males, were used for the present study. Throughout these studies, all of the mice were maintained on a non-restricted, regular (19%) protein diet (PicoLab High Energy Mouse Diet from LabDiet) and without the administration of ammonia scavengers. Mice were injected with an adenovirus vector expressing urokinase plasminogen activator ∼24 h before transplantation (Yecuris, Tualatin, OR, USA).[14,15] Cells were transplanted directly into the spleen of the mice under isoflurane anesthesia (Baxter, Norfolk, UK). Approximately 1×10^6 live hepatocytes were transplanted in each mouse in 200 μL plasmalyte. The cells were injected slowly over an ∼1-min period. Animals received analgesic treatment immediately after and 24 h post-surgery. Mice were cycled on and off the protective drug 2-(2-nitro-4-trifluoromethyl-benzoyl)-1,3-cyclohexanedione (NTBC) (Sobi, Stockholm, Sweden) to stimulate the expansion of donor human hepatocytes. Specifically, before hepatocyte transplantation, NTBC was provided at a concentration of 16 mg/L in the drinking water. From the day of transplantation until day 65 post-transplantation, mice were cycled 7 days off NTBC and 3 days on 8 mg/L NTBC, and after day 65 they were cycled 21 days off NTBC and 2–3 days on 8 mg/L NTBC.

Three experimental groups were included in the present study, mice injected with unedited OTCD primary hepatocytes (unedited OTCD group), mice injected with genetically modified OTCD hepatocytes (edited OTCD group), and those that received OTCP hepatocytes (OTCP group). Fourteen mice were transplanted with unedited OTCD hepatocytes, 9 of which survived the transplantation procedure, reached the desired repopulation level, and eventually were included in the study (9/14, 64%). In the edited OTCD group, 27 mice received edited hepatocytes, 18 of which were fully humanized and included in the study (18/27, 67%). In addition, 30 mice were injected with OTCP cells, and of those, 17 were fully humanized and included in the study (17/30, 57%). Mice that were excluded from the study either failed to reach targeted levels of repopulation or had to be euthanized because of unacceptable loss of weight as required by our approved animal protocols. Animals were 6 weeks old at the time of transplantation. They reached the aimed-for circulating human albumin on average 3–4 months post-transplantation, and consequently the intended levels of liver humanization. At that time point, basal and post-injection (ammonia challenge) ammonia levels were evaluated. Ultimately, animals were euthanized, and other assays were carried out (e.g., enzyme activities, gene expression, immunohistochemistry).

Blood sampling and ELISA

Human albumin concentrations in mouse blood were monitored approximately twice per month starting from the 4th to the 5th week post-transplantation. Blood 2 μL was diluted in 198 μL diluent and analyzed with the Quantitative Human Albumin ELISA Quantitation Kit (Bethyl Laboratory, Montogmery, TX, USA). The level of humanization was estimated based on previous studies in which each 1 mg/mL of circulating human albumin corresponds to an ∼20% level of repopulation of the murine liver with human hepatocytes.[14,15] Circulating human A1AT levels were estimated with the Human alpha 1 Antritrypsin ELISA Kit (SERPINA1) (Abcam), according to the manufacturer's instructions.

Ammonia measurement and ureagenesis

Blood ammonia measurements were made on mice that received transplants with unedited OTCD hepatocytes when they had an estimated level of repopulation ≥80% (≥4 mg/mL serum human albumin). Mice that were transplanted with edited OTCD hepatocytes or normal OTCP hepatocytes were analyzed when they were estimated to be fully repopulated (≥5 mg/mL serum human albumin). A different albumin threshold and lower level of repopulation of the liver were used for mice that received unedited OTCD human

hepatocytes to prevent the most severe symptoms of OTCD, including severe hyperammonemia, lethargy, and weight loss.

Animals were off NTBC at least 4 days before the ureagenesis assays were conducted. Animals in the edited group had been off NTBC for 18.5 days on average before the analysis of ureagenesis. Blood ammonia levels were measured with Arkray Pocket Chem (Arkray) by diluting 5 or 10 μL of blood in 20 μL water and following the manufacturer's instructions. Ammonia was measured before and 30 min after intraperitoneal infusion of 4 mmol/kg body weight NH_4Cl (Sigma-Aldrich), and are reported here as "basal ammonia" and "post-injection ammonia," respectively.

Determination of urinary orotic acid

Orotic acid was determined by diluting 10 μL freshly frozen urine with 190 μL 11 mM NH_4OH solution. These samples were then incubated for 30 min at 37°C and subsequently centrifuged at room temperature for 10 min at 10,000 rpm. The supernatant (150 μL) was transferred into a liquid chromatography (LC) vial together with 130 μL 0.1% formic acid and 20 μL internal standard (1,3-$^{15}N_2$-orotic acid, final concentration 6 μM) ready for analysis. Samples were measured using a Thermo Ultimate 3000 UHPLC (Thermo Scientific, Olten, Switzerland) interfaced to an AB SCIEX Triple Quad 5500 mass spectrometer with a TurboV electrospray ionization source (AB Sciex, Zug, Switzerland). Separation was achieved using a reverse-phase ACQUITY UPLC HSS T3 1.8 μm (2.1 × 50 mm, Waters, Baden, Switzerland) with a matching T3 VanGuard pre-column, with mobile phase A and B consisting of 2 mM ammonium formate in 0.1% formic acid and MeOH, respectively. The analysis of orotic acid was performed by injecting 2 μL of the prepared sample using a flow rate of 300 μL/min and a column temperature of 20°C. Orotic acid and its internal standard were analyzed using the multiple reaction monitoring (MRM) pairs 155.1 →111.1 and 157.1 → 113.1, respectively, with the following compound parameters: DE clustering potential −50 V, entrance potential −10 V, collision energy −11 V, and collision cell exit potential −11 V, with a dwell time of 20 ms. Ion source parameters were as follows: IonSpray voltage −3.5 kV, temperature 500°C, curtain gas 25, and collision gas 10. All of the parameters were optimized by infusion experiments in the negative ionization mode. The quantification was completed using a standard calibration curve in the MultiQuant 3.0.2 software (AB Sciex, Zug, Switzerland). Orotic acid values in samples were normalized to their respective creatinine levels.

Liver OTC and CPS1 enzyme activities

Liver extracts and OTC and CPS1 analysis was conducted as described by Allegri et al.[30] In brief, an assay mixture with a total volume of 300 μL (pH 7.7) containing a final concentration of triethanolamine (270 mM), ornithine (2.5 mM), and carbamoyl phosphate (5.8 mM) was prepared, and a volume of 25 μL liver lysate, equivalent to 0.25–10 μg total protein, was added. The reaction mixture was incubated at 37°C for 30 min, and the reaction was stopped by adding 0.7 mL of a 2:1 solution of 19.65 mM antipyrine, 5.18 mM iron (III) ammonium sulfate, 25% phosphoric acid and 25% sulfuric acid, and 1.28 M NaCl and 39.56 mM 2,3-butanedione monoxime. The reactional mixture was incubated at 95°C–100°C for 15 min in the dark. The production of citrulline was determined and quantified at 464 nm with a citrulline standard curve.

CPS1 activity was assayed in liver lysates at 37°C for 11 min in a coupled reaction, in which the carbamoyl phosphate formed was immediately converted to citrulline from OTC, as previously described.[31]

Gene expression analysis

Liver was collected from mice that received unedited or edited OTCD hepatocytes, as well as hepatocytes from a normal OTCP donor. Portions of the organ were fixed in formalin for histological analysis and the remaining portion was crushed and mixed to a homogeneous powder under liquid nitrogen. RNA was extracted using TRIzol solution (Thermo Fisher) and cDNA was synthesized with a high-capacity RNA-to-cDNA kit (Thermo Fisher). TaqMan assays (Thermo Fisher) were used to quantify gene expression, and cyclophilin A (*PPIA*) served as the endogenous control for the normalization of the obtained Ct values. All of the assays used were checked for cross-reactivity between human and mouse transcriptome. Liver tissue from untransplanted FRGN mice was used as the negative control for the gene expression analysis. Details of the TaqMan assays are provided in Table S2.

Histology

Liver sections from animals repopulated with edited OTCD, unedited OTCD, or OTCP hepatocytes were collected for histological analysis. Briefly, tissues were fixed in 4% buffered formalin, embedded in paraffin, and sectioned to a 4-μM thickness. The liver tissues were stained with human-specific antibodies for anti-cytokeratins 8 and 18 (clone 5D3, dilution 1:100; Thermo Fisher) or anti-OTC (HPA000243, dilution 1:400; Sigma-Aldrich), and counterstained with hematoxylin. Pictures were taken with an Olympus IX73 inverted microscope and analyzed with Cleans Dimension 1.12 software.

CIRCLE-seq

CIRCLE-seq was performed experimentally, as previously described.[18] Data were processed using version 1.1 of the CIRCLE-Seq analysis pipeline (https://github.com/tsailabSJ/circleseq) with parameters "window_size: 3; mapq_threshold: 50; start_threshold: 1; gap_threshold: 3; mismatch_threshold: 7; merged_analysis: False, variant_analysis: True." Gene annotations were identified using Ensembl hg38 version 92.

Targeted amplicon deep sequencing for off-target evaluation

Genomic DNA from edited hepatocytes was extracted using the Gentra Puregene Tissue kit (QIAGEN). All of the off-target sites analyzed were amplified from 80 ng of input genomic DNA with Q5 High-Fidelity DNA polymerase (New England Biolabs) using amplicon-specific primers attached with sequencing adapters (5′′

TCGTCGGCAGCGTCAGATGTGTATAAGAGACAG-[amplicon specific primer]-3′ and 5′-GTCTCGTGGGCTCG GAGATGTGTA-TAAGAGACAG-[amplicon specific primer]-3′). Amplicon-specific primers are listed in Tables S5 and S6. PCR products were purified and size selected using magnetic beads (Agencourt AMPure XP), quantified using fragment analyzer (Advanced Analytical Technologies), and amplicons of 6–10 sites were pooled to be equimolar. Pooled PCR products were indexed (Illumina Nextera XT index kit) and purified with magnetic beads. Final libraries were quantified using Qubit Fluorometer (Thermo Fisher) and loaded onto an Illumina NextSeq for deep sequencing. Amplicon sequencing data were analyzed using CRISPResso2 software (https://github.com/pinellolab/crispresso2),[32] with the following parameters: -q 30–ignore_substitutions–max_paired_end_read-s_overlap 200 -w 1. For each of the sites we examined, we obtained $\geq$10,000 sequencing reads, except that site 14 for gRNA5 had 3 samples with 8,459, 9,798, and 9,973 reads.

Statistical analysis of targeted amplicon deep sequencing data

p values were obtained by fitting a generalized linear model with a Poisson distribution (function glm in R version 3.6.0 and with the logarithm of the total number of reads as the offset) to the control and edited samples for each evaluated site. We adjusted for multiple comparisons using the Benjamini-Hochberg method (function p.adjust in R version 3.6.0). We considered the indel percentage in the gRNA/Cas9-transfected replicates to be significantly greater than the indel percentage in the Cas9-transfected controls if the adjusted p value was <0.1, the nuclease-treatment coefficient was >0, and the median indel frequency of the edited replicates was >0.1%. If there was no significant difference between control and gRNA treated samples, then this variation was not attributed to Cas9 nuclease activity. An r function to execute this analysis can be found at https://github.com/nvanzuy/crispr_otc_analysis.

Statistical analysis

All of the statistical analysis were performed using GraphPad Prism. Sample size (n) is indicated in the relevant figure legends. Ordinary ANOVA and Tukey multiple comparison tests or Kruskal-Wallis ANOVA and Dunn's multiple comparison tests were used to compare means between three experimental groups, normally distributed or not, respectively. A two-tailed, unpaired t test or a Mann-Whitney *U* test was used to compare means between two experimental groups, normally distributed or not, respectively. The statistical analysis of targeted amplicon deep sequencing data is described in the respective method section. Group averages and errors are shown as medians and interquartile ranges. The level of significance was set at $p < 0.05$ for all of the experiments (summarized as *p > 0.05; **p < 0.01; ***p < 0.001; ****p < 0.0001).

SUPPLEMENTAL INFORMATION

Supplemental information can be found online at https://doi.org/10.1016/j.ymthe.2021.01.024.

ACKNOWLEDGMENTS

This research was supported by the Center for Innovative Medicine (CIMED), Sweden (grant no. 3115), Vetenskapsrådet (Swedish Research Council), Sweden (grant no. 2017-02789), the European Commission EU/FP7 - Health and the Understanding of Metabolism, Aging and Nutrition (HUMAN; grant no. 602757), and the Torsten och Ragnar Söderberg Stiftelse, Sweden (grant no. 2013-1269) to S.C.S.. Additional support was provided by AstraZeneca, Sweden. Part of the work was supported by the Swiss National Science Foundation, Switzerland (grant no. 320030_176088) to J.H.

AUTHOR CONTRIBUTIONS

M.Z. designed and performed the experiments, analyzed the data, and wrote the manuscript. R.C.S. and C.H. performed the experiments and analyzed the data. P.A., B.B., M.F., N.R.v.Z., and M.M. performed the CIRCLE-seq experiments and the targeted deep-sequencing experiments, analyzed the data, and reviewed the manuscript. G.A., G.M., T.S., and N.R. analyzed the enzyme activities and orotic acid. T.J. conducted the *in vitro* ureagenesis measurements. E.E. and C.J. assisted with the primary hepatocyte isolation and the experiments. G.A., T.J., R.G., A.F., and S.E. reviewed the manuscript. O.R., B.T., J.H., and M.M. reviewed the manuscript and supervised the experiments. B.R. designed the experiments and wrote the manuscript. S.C.S. designed and supervised the experiments and wrote the manuscript. All of the authors approved the final version.

DECLARATION OF INTERESTS

S.C.S. has stock in Yecuris (no Yecuris animals were used in these studies).

REFERENCES

1. Summar, M.L., Koelker, S., Freedenberg, D., Le Mons, C., Haberle, J., Lee, H.S., and Kirmse, B.; European Registry and Network for Intoxication Type Metabolic Diseases (E-IMD); Members of the Urea Cycle Disorders Consortium (UCDC) (2013). The incidence of urea cycle disorders. Mol. Genet. Metab. *110*, 179–180.
2. Fox, I.J., Chowdhury, J.R., Kaufman, S.S., Goertzen, T.C., Chowdhury, N.R., Warkentin, P.I., Dorko, K., Sauter, B.V., and Strom, S.C. (1998). Treatment of the Crigler-Najjar syndrome type I with hepatocyte transplantation. N. Engl. J. Med. *338*, 1422–1426.
3. Fisher, R.A., and Strom, S.C. (2006). Human hepatocyte transplantation: worldwide results. Transplantation *82*, 441–449.
4. Häberle, J., Burlina, A., Chakrapani, A., Dixon, M., Karall, D., Lindner, M., Mandel, H., Martinelli, D., Pintos-Morell, G., Santer, R., et al. (2019). Suggested guidelines for the diagnosis and management of urea cycle disorders: first revision. J. Inherit. Metab. Dis. *42*, 1192–1230.
5. Soltys, K.A., Setoyama, K., Tafaleng, E.N., Soto Gutiérrez, A., Fong, J., Fukumitsu, K., Nishikawa, T., Nagaya, M., Sada, R., Haberman, K., et al. (2017). Host conditioning and rejection monitoring in hepatocyte transplantation in humans. J. Hepatol. *66*, 987–1000.
6. Jorns, C., Nowak, G., Nemeth, A., Zemack, H., Mörk, L.M., Johansson, H., Gramignoli, R., Watanabe, M., Karadagi, A., Alheim, M., et al. (2016). De Novo Donor-Specific HLA Antibody Formation in Two Patients With Crigler-Najjar Syndrome Type I Following Human Hepatocyte Transplantation With Partial Hepatectomy Preconditioning. Am. J. Transplant. *16*, 1021–1030.
7. Cong, L., Ran, F.A., Cox, D., Lin, S., Barretto, R., Habib, N., Hsu, P.D., Wu, X., Jiang, W., Marraffini, L.A., and Zhang, F. (2013). Multiplex genome engineering using CRISPR/Cas systems. Science *339*, 819–823.

8. Doudna, J.A., and Charpentier, E. (2014). Genome editing. The new frontier of genome engineering with CRISPR-Cas9. Science *346*, 1258096.

9. Mali, P., Yang, L., Esvelt, K.M., Aach, J., Guell, M., DiCarlo, J.E., Norville, J.E., and Church, G.M. (2013). RNA-guided human genome engineering via Cas9. Science *339*, 823–826.

10. Yin, H., Xue, W., Chen, S., Bogorad, R.L., Benedetti, E., Grompe, M., Koteliansky, V., Sharp, P.A., Jacks, T., and Anderson, D.G. (2014). Genome editing with Cas9 in adult mice corrects a disease mutation and phenotype. Nat. Biotechnol. *32*, 551–553.

11. Yin, H., Song, C.Q., Dorkin, J.R., Zhu, L.J., Li, Y., Wu, Q., Park, A., Yang, J., Suresh, S., Bizhanova, A., et al. (2016). Therapeutic genome editing by combined viral and non-viral delivery of CRISPR system components in vivo. Nat. Biotechnol. *34*, 328–333.

12. Yang, Y., Wang, L., Bell, P., McMenamin, D., He, Z., White, J., Yu, H., Xu, C., Morizono, H., Musunuru, K., et al. (2016). A dual AAV system enables the Cas9-mediated correction of a metabolic liver disease in newborn mice. Nat. Biotechnol. *34*, 334–338.

13. Villiger, L., Grisch-Chan, H.M., Lindsay, H., Ringnalda, F., Pogliano, C.B., Allegri, G., Fingerhut, R., Häberle, J., Matos, J., Robinson, M.D., et al. (2018). Treatment of a metabolic liver disease by in vivo genome base editing in adult mice. Nat. Med. *24*, 1519–1525.

14. Azuma, H., Paulk, N., Ranade, A., Dorrell, C., Al-Dhalimy, M., Ellis, E., Strom, S., Kay, M.A., Finegold, M., and Grompe, M. (2007). Robust expansion of human hepatocytes in Fah-/-/Rag2-/-/Il2rg-/- mice. Nat. Biotechnol. *25*, 903–910.

15. Wilson, E.M., Bial, J., Tarlow, B., Bial, G., Jensen, B., Greiner, D.L., Brehm, M.A., and Grompe, M. (2014). Extensive double humanization of both liver and hematopoiesis in FRGN mice. Stem Cell Res. (Amst.) *13* (3 Pt A), 404–412.

16. Srinivasan, R.C., Zabulica, M., Hammarstedt, C., Wu, T., Gramignoli, R., Kannisto, K., Ellis, E., Karadagi, A., Fingerhut, R., Allegri, G., et al. (2019). A liver-humanized mouse model of carbamoyl phosphate synthetase 1-deficiency. J. Inherit. Metab. Dis. *42*, 1054–1063.

17. Ogino, W., Takeshima, Y., Nishiyama, A., Okizuka, Y., Yagi, M., Tsuneishi, S., Saiki, K., Kugo, M., and Matsuo, M. (2007). Mutation analysis of the ornithine transcarbamylase (OTC) gene in five Japanese OTC deficiency patients revealed two known and three novel mutations including a deep intronic mutation. Kobe J. Med. Sci. *53*, 229–240.

18. Tsai, S.Q., Nguyen, N.T., Malagon-Lopez, J., Topkar, V.V., Aryee, M.J., and Joung, J.K. (2017). CIRCLE-seq: a highly sensitive in vitro screen for genome-wide CRISPR-Cas9 nuclease off-targets. Nat. Methods *14*, 607–614.

19. Akcakaya, P., Bobbin, M.L., Guo, J.A., Malagon-Lopez, J., Clement, K., Garcia, S.P., Fellows, M.D., Porritt, M.J., Firth, M.A., Carreras, A., et al. (2018). In vivo CRISPR editing with no detectable genome-wide off-target mutations. Nature *561*, 416–419.

20. Ginn, S.L., Amaya, A.K., Liao, S.H.Y., Zhu, E., Cunningham, S.C., Lee, M., Hallwirth, C.V., Logan, G.J., Tay, S.S., Cesare, A.J., et al. (2019). Efficient *in vivo* editing of OTC-deficient patient-derived primary human hepatocytes. JHEP Rep *2*, 100065.

21. Kosicki, M., Tomberg, K., and Bradley, A. (2018). Repair of double-strand breaks induced by CRISPR-Cas9 leads to large deletions and complex rearrangements. Nat. Biotechnol. *36*, 765–771.

22. Nelson, C.E., Wu, Y., Gemberling, M.P., Oliver, M.L., Waller, M.A., Bohning, J.D., Robinson-Hamm, J.N., Bulaklak, K., Castellanos Rivera, R.M., Collier, J.H., et al. (2019). Long-term evaluation of AAV-CRISPR genome editing for Duchenne muscular dystrophy. Nat. Med. *25*, 427–432.

23. Irani, A.N., Malhi, H., Slehria, S., Gorla, G.R., Volenberg, I., Schilsky, M.L., and Gupta, S. (2001). Correction of liver disease following transplantation of normal rat hepatocytes into Long-Evans Cinnamon rats modeling Wilson's disease. Mol. Ther. *3*, 302–309.

24. Ding, J., Yannam, G.R., Roy-Chowdhury, N., Hidvegi, T., Basma, H., Rennard, S.I., Wong, R.J., Avsar, Y., Guha, C., Perlmutter, D.H., et al. (2011). Spontaneous hepatic repopulation in transgenic mice expressing mutant human α1-antitrypsin by wild-type donor hepatocytes. J. Clin. Invest. *121*, 1930–1934.

25. De Vree, J.M.L., Ottenhoff, R., Bosma, P.J., Smith, A.J., Aten, J., and Oude Elferink, R.P.J. (2000). Correction of liver disease by hepatocyte transplantation in a mouse model of progressive familial intrahepatic cholestasis. Gastroenterology *119*, 1720–1730.

26. Wang, X., Raghavan, A., Chen, T., Qiao, L., Zhang, Y., Ding, Q., and Musunuru, K. (2016). CRISPR-Cas9 Targeting of PCSK9 in Human Hepatocytes In Vivo-Brief Report. Arterioscler. Thromb. Vasc. Biol. *36*, 783–786.

27. Vosough, M., Ravaioli, F., Zabulica, M., Capri, M., Garagnani, P., Franceschi, C., Piccand, J., Kraus, M.R., Kannisto, K., Gramignoli, R., and Strom, S.C. (2019). Applying hydrodynamic pressure to efficiently generate induced pluripotent stem cells via reprogramming of centenarian skin fibroblasts. PLoS ONE *14*, e0215490.

28. Rivera-Barahona, A., Sanchez-Alcudia, R., Viecelli, H.M., Rufenacht, V., Perez, B., Ugarte, M., et al. (2015). Functional Characterization of the spf/ash Splicing Variation in OTC Deficiency of Mice and Man. PLoS ONE *10*, e0122966.

29. Allegri, G., Deplazes, S., Grisch-Chan, H.M., Mathis, D., Fingerhut, R., Häberle, J., and Thöny, B. (2017). A simple dried blood spot-method for in vivo measurement of ureagenesis by gas chromatography-mass spectrometry using stable isotopes. Clin. Chim. Acta *464*, 236–243.

30. Allegri, G., Deplazes, S., Rimann, N., Causton, B., Scherer, T., Leff, J.W., Diez-Fernandez, C., Klimovskaia, A., Fingerhut, R., Krijt, J., et al. (2019). Comprehensive characterization of ureagenesis in the spf^{ash} mouse, a model of human ornithine transcarbamylase deficiency, reveals age-dependency of ammonia detoxification. J. Inherit. Metab. Dis. *42*, 1064–1076.

31. Diez-Fernandez, C., Martínez, A.I., Pekkala, S., Barcelona, B., Pérez-Arellano, I., Guadalajara, A.M., Summar, M., Cervera, J., and Rubio, V. (2013). Molecular characterization of carbamoyl-phosphate synthetase (CPS1) deficiency using human recombinant CPS1 as a key tool. Hum. Mutat. *34*, 1149–1159.

32. Clement, K., Rees, H., Canver, M.C., Gehrke, J.M., Farouni, R., Hsu, J.Y., Cole, M.A., Liu, D.R., Joung, J.K., Bauer, D.E., and Pinello, L. (2019). CRISPResso2 provides accurate and rapid genome editing sequence analysis. Nat. Biotechnol. *37*, 224–226.

YMTHE, Volume *29*

Supplemental Information

Correction of a urea cycle defect after *ex vivo* gene editing of human hepatocytes

Mihaela Zabulica, Raghuraman C. Srinivasan, Pinar Akcakaya, Gabriella Allegri, Burcu Bestas, Mike Firth, Christina Hammarstedt, Tomas Jakobsson, Towe Jakobsson, Ewa Ellis, Carl Jorns, Georgios Makris, Tanja Scherer, Nicole Rimann, Natalie R. van Zuydam, Roberto Gramignoli, Anna Forslöw, Susanna Engberg, Marcello Maresca, Olav Rooyackers, Beat Thöny, Johannes Häberle, Barry Rosen, and Stephen C. Strom

Supplemental Figures

A

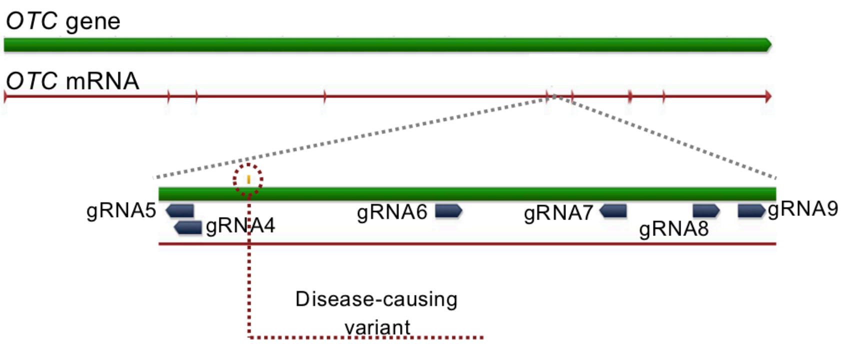

B

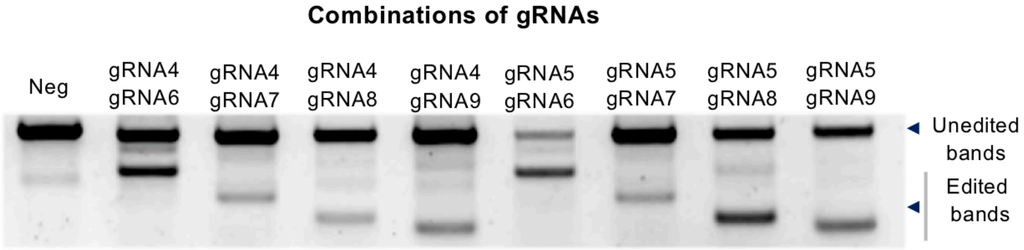

Supplemental Figure S1: Screening of combinations of gRNAs for efficient cleavage of the ectopic splice site. (A) Genomic structure representation of *OTC* gene (green) and transcript (red), and the position of CRISPR gRNAs tested. Two alternatives of gRNAs are positioned upstream (gRNA4 and gRNA5), and four gRNAs downstream (gRNA6, gRNA7, gRNA8 and gRNA9) of the pathogenic variant, and are used pairwise. **(B)** Estimation of editing efficiency of different pairs of gRNAs by PCR amplification of the target region. Upper and lower amplicon bands show unedited (wild-type) and edited bands, respectively, and are indicated with arrows. Neg: Non-transfected cells.

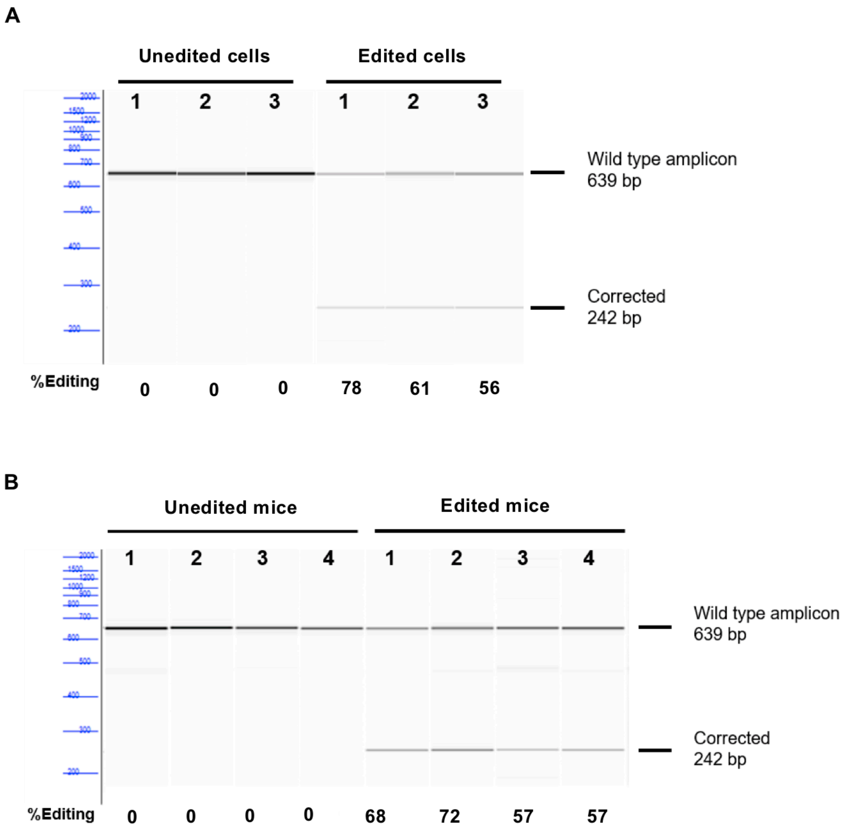

Supplemental Figure S2: gRNA5-gRNA8 dual guides with *Sp*Cas9 efficiently mutates *OTC* gene in patient-derived primary hepatocytes and humanized mouse livers. (A) Editing was evaluated in n = 3 transfection replicates (labelled as 1, 2 and 3) using genomic DNA isolated from the cells electroporated with dual RNP complex gRNA5-Cas9 and gRNA8–Cas9, and unedited OTCD cells electroporated with only Cas9 enzyme. Assay was performed 48 hours following electroporation. **(B)** Editing was evaluated in n = 4 biological replicates (labelled as 1, 2, 3 and 4) using genomic DNA isolated from the humanized mouse livers transplanted with corrected patient hepatocytes edited by dual RNP complex gRNA5-Cas9 and gRNA8–Cas9, and unedited OTCD cells. Assay was performed when mice reached desired repopulation levels. Gene region was amplified with sequence specific primers. Wild type (639 bp) and mutated (242 bp) amplicons were quantified using fragment analyzer.

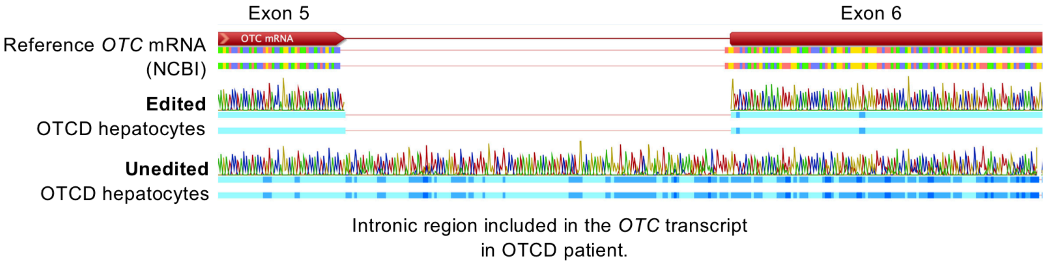

Supplemental Figure S3: Sequencing of *OTC* transcript in edited and unedited OTC deficient (OTCD) hepatocytes. *OTC* transcripts in genetically edited and unedited patient OTCD hepatocytes were amplified, sequenced and aligned to reference *OTC* transcript (NCBI NP_000522.3). The intronic region included between exons 5 and 6 in the mutated transcript in patient OTCD hepatocytes is absent in OTCD edited cells.

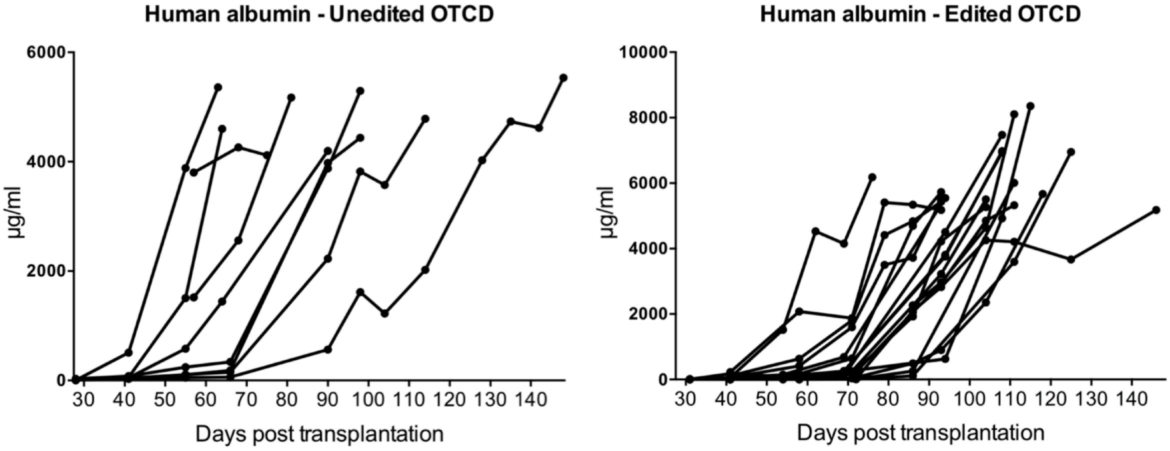

Supplemental Figure S4: Human albumin levels at different time points post transplantation in Unedited OTC deficient (OTCD) and Edited OTCD groups. Each line represents an experimental animal. Unedited OTCD mice n=9. Edited OTCD mice n=18.

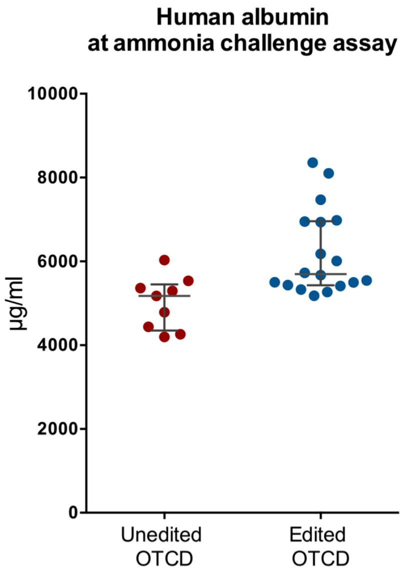

Supplemental Figure S5: Human albumin levels at ammonia challenge. Human albumin measurements when mice were subjected to ammonia challenge assay *in vivo.* Unedited OTCD mice n=9. Edited OTCD mice n=18.

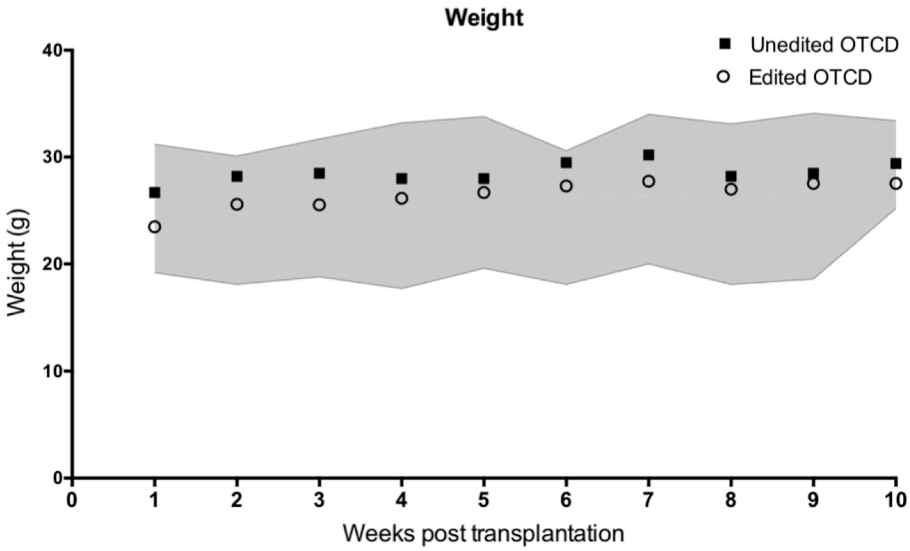

Supplemental Figure S6: Weight. Median weight of experimental groups that were either liver-humanized with edited or unedited patient OTC deficient (OTCD) hepatocytes every week post transplantation. Grey-highlighted area represents weight range of healthy controls (mice repopulated with OTC proficient hepatocytes).

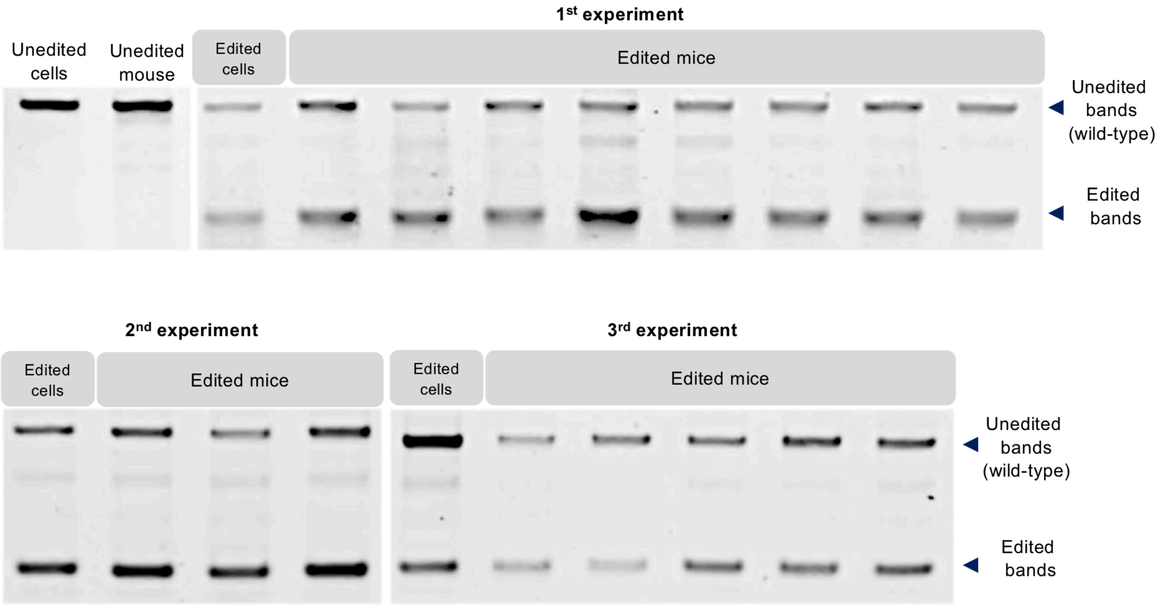

Supplemental Figure S7: Estimation of editing efficiency in hepatocytes prior transplantation and in liver-humanized mice with the respective cells. PCR amplification of target region in the *OTC* gene in OTC deficient (OTCD, either edited or unedited) hepatocytes prior transplantation and in humanized livers with the respective cells from three independent experiments. Upper and lower band indicate unedited (wild-type) and edited DNA, respectively. Actual editing efficiency was estimated based on band intensity and amplicon length, and shown in Figure 3A. Unedited cells: OTCD hepatocytes not genetically engineered. Unedited mouse: Humanized mouse liver with unedited OTCD hepatocytes. Edited cells: OTCD hepatocytes genetically engineered and used for each experiment. Edited mice: Humanized mouse livers with edited OTCD hepatocytes (each lane corresponds to DNA from a different humanized mouse liver).

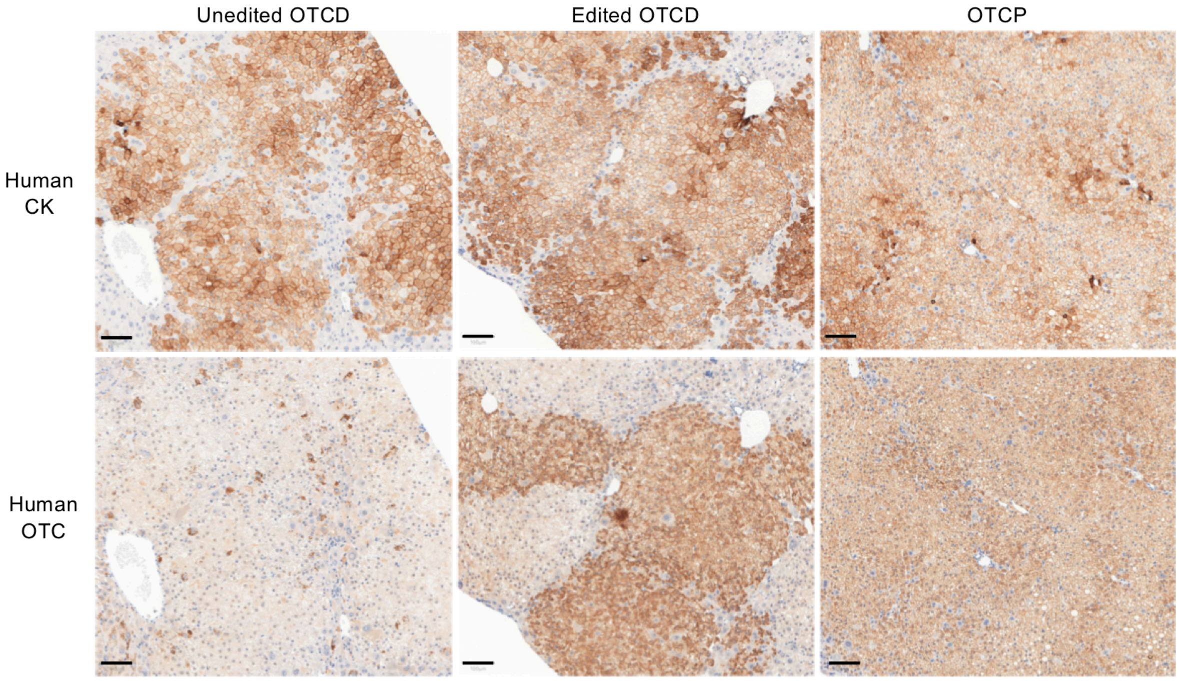

Supplemental Figure S8: Immunohistochemical analyses of liver tissue from liver-humanized mice. Representative sections of mouse liver immunostained with antibodies against human CK or OTC in mice that received either unedited or edited OTC deficient (OTCD), or OTC proficient (OTCP) human hepatocytes. Areas positive for CK indicate the areas repopulated with human hepatocytes. Positive reaction with antibodies to OTC indicates cells that express full-length, normal protein. Scale bar 100 µm.

Supplemental Tables

Supplemental Table 1: Sequence and position of gRNA tested for optimizing editing efficiency.

gRNA name	5' - sequence - 3'
gRNA4	GATATGGGCTAAAAGGGTTT
gRNA5	GGCTAAAAGGGTTTGGGAAT
gRNA6	GGTTAGTTACTAAGTCACTC
gRNA7	CTAATTTGGGAGGACTAGAA
gRNA8	AATCTACATTATATTGCCCC
gRNA9	AGCCATGGTTTCTATGTCTA

Position of gRNAs tested:

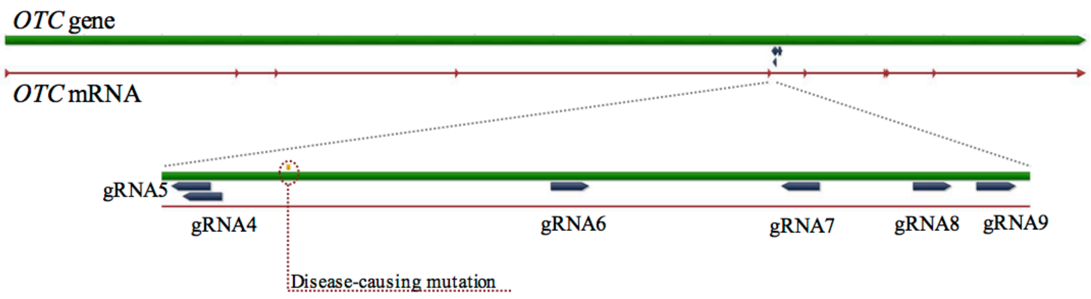

Supplemental Table 2: TaqMan assays used for gene expression analyses.

	Gene name	Gene full name	Assay ID
	PPIA	Cyclophilin A (peptidylprolyl isomerase A)	Hs99999904_m1
**Urea cycle genes**	*OTC*	Ornithine carbamoyltransferase	Hs00166892_m1
	CPS1	Carbamoyl-phosphate synthetase 1, mitochondrial	Hs00157048_m1
	ASS1	Argininosuccinate synthetase	Hs01597989_g1
	ASL	Argininosuccinate lyase	Hs00902699_m1
	ARG1	Arginase 1	Hs00163660_m1
**Liver proteins and transcription factors**	*ALB*	Albumin	Hs00609411_m1
	AFP	Alpha-fetoprotein	Hs00173490_m1
	FAH	Fumarylacetoacetate hydrolase	Hs00908445_m1
	A1AT	Alpha-1 antitrypsin (SERPINA1)	Hs01097800_m1
	HNF4a	Hepatic nuclear factor 4 alpha	Hs0102395_m1
	HNF6	Hepatic nuclear factor 6 (ONECUT1)	Hs00413554_m1
	CAR	Nuclear receptor subfamily 1, I3 (NR1I3)	Hs00901570_g1
	PXR	Nuclear receptor subfamily 1, I2 (NR1I2)	Hs01114267_m1
**Phase I genes**	*CYP1A1*	Cytochrome P450 family 1 subfamily A member 1	Hs00153120_m1
	CY1A2	Cytochrome P450 family 1 subfamily A member 2	Hs01070374_m1
	CYP2B6	Cytochrome P450 family 2 subfamily B member 6	Hs03044634_m1
	CYP2C8	Cytochrome P450 family 2 subfamily C member 8	Hs00258314_m1
	CYP2C9	Cytochrome P450 family 2 subfamily C member 9	Hs00426397_m1
	CYP2C19	Cytochrome P450 family 2 subfamily C member 19	Hs00426380_m1
	CYP3A4	Cytochrome P450 family 3 subfamily A member 4	Hs00430021_m1
	CYP3A7	Cytochrome P450 family 3 subfamily A member 7	Hs00426361_m1
**Phase II genes**	*UGT1A1*	UDP glucuronosyltransferase 1 family, A1	Hs02511055_s1
	UGT1A6	UDP glucuronosyltransferase 1 family, A6	Hs01592477_m1
	UGT1A9	UDP glucuronosyltransferase 1 family, A9	Hs02516855_sH
	UGT2B7	UDP glucuronosyltransferase 2 family, B7	Hs00426592_m1
	UGT2B17	UDP glucuronosyltransferase 2 family, B17	Hs00854486_sH
**Transporters**	*MRP2*	ATP-binding cassette, C2 (ABCC2)	Hs00166123_m1
	MRP3	ATP-binding cassette, C3 (ABCC3)	Hs00978473_m1
	MRP4	ATP-binding cassette, C4 (ABCC4)	Hs00988717_m1
	BSEP	ATP-binding cassette, B11 (ABCB11)	Hs00184824_m1
	BCRP	ATP-binding cassette, G2 (ABCG2)	Hs00184979_m1
	NTCP	Sodium/bile acid cotransporter 1 (SLC10A1)	Hs00914889_m1

Supplemental Table 3: Provided as a separate Excel file.
Off-target sites identified by CIRCLE-seq for gRNA5 and Cas9 nuclease. Chromosomal coordinates are listed, followed by the CIRCLE-seq read count for each site for two replicates, the off-target sequence, number of mismatches, bulge mismatch sequence, score for the off-targets with bulges, and annotations according to Ensembl as gene and region.

Supplemental Table 4: Provided as a separate Excel file.
Off-target sites identified by CIRCLE-seq for gRNA8 and Cas9 nuclease. Chromosomal coordinates are listed, followed by the CIRCLE-seq read count for each site for two replicates, the off-target sequence, number of mismatches, bulge mismatch sequence, score for the off-targets with bulges, and annotations according to Ensembl as gene and region.

Supplemental Table 5: Provided as a separate Excel file.
Potential off-target sites for gRNA5 and Cas9, analysed by targeted amplicon deep sequencing in primary hepatocytes. Samples are organized according to treatment status and replicate number. For each off-target site, chromosomal coordinates are listed, followed by columns detailing the read counts for each sample, median percentage of indels for each treatment group and statistical test results between the edited and control groups, followed by primers used. Coef_flag: Nuclease-treatment coefficient, freq_flag: median indel frequency of edited samples and sig_flag: adjusted p value are evaluated to determine off-targets reported in the off-target column.

Supplemental Table 6: Provided as a separate Excel file.
Potential off-target sites for gRNA8 and Cas9, analyzed by targeted amplicon deep sequencing in primary hepatocytes. Samples are organized according to treatment status and replicate number. For each off-target site, chromosomal coordinates are listed, followed by columns detailing the read counts for each sample, median percentage of indels for each treatment group and statistical test results between the edited and control groups, followed by primers used. Coef_flag: Nuclease-treatment coefficient, freq_flag: median indel frequency of edited samples and sig_flag: adjusted p value are evaluated to determine off-targets reported in the off-target column.
